# Analysis of choroidal features to predict surgical prognosis of idiopathic macular hole

**DOI:** 10.1371/journal.pone.0308292

**Published:** 2024-09-06

**Authors:** Gee-Hyun Kim, Jiho Lee, Young-Hoon Park

**Affiliations:** 1 Department of Ophthalmology, Seoul St. Mary’s Hospital, College of Medicine, The Catholic University of Korea, Seoul, Republic of Korea; 2 Catholic Institute for Visual Science, College of Medicine, The Catholic University of Korea, Seoul, Republic of Korea; Hangil Eye Hospital / Catholic Kwandong University College of Medicine, REPUBLIC OF KOREA

## Abstract

**Objective:**

To investigate choroidal features of idiopathic macular hole (IMH) and determine their relationship with surgical outcomes.

**Materials and methods:**

Patients above stage II unilateral IMH who received pars plana vitrectomy (PPV) with the internal limiting membrane (ILM) peeling were enrolled for the retrospective observational study. Preoperative choriocapillaris perfusion (CCP), central choroidal thickness (CCT), base/minimum diameters (BD/MD) and height (HH) of MH were analyzed by optical coherence tomography angiography (OCTA). At 1, 3 and 6 months after PPV, CCT, central foveal thickness (CFT) and maximum parafoveal thickness (MPT) of closed MH were measured. Best-corrected visual acuity (BCVA) was assessed at every visit. The correlations between preoperative characteristics and surgical outcomes were assessed.

**Results:**

Twenty-seven patients were evaluated. All eyes (100%) showed successful MH closure after the primary surgery. Until postoperative 6 months, BCVA continued to improve significantly (*p* < 0.001), while CFT and CCT progressively thinned (*p* < 0.001, *p* < 0.001). On correlation tests, final postoperative BCVA was associated with preoperative BCVA (R = 0.506, *p* = 0.007) and CCP (R = -0.475, *p* = 0.012), while final CFT was related with preoperative CCT (R = 0.392, *p* = 0.043). Multiple regression analysis revealed that preoperative CCP was significantly related with final postoperative BCVA (β = -0.403, p = 0.049).

**Conclusion:**

Preoperative CCP and CCT were respectively associated with functional and anatomical prognosis of IMH after PPV.

## Introduction

A macular hole (MH) is a retinal defect in the macula from internal limiting membrane (ILM) to the outer segment of photoreceptor layer [1]. MH may be associated with high myopia or caused by ocular trauma, but most cases are idiopathic [1]. The cause of idiopathic macular hole (IMH) has been suggested as vitreofoveal traction and tangential traction of ILM, but the exact pathogenesis is still uncertain [1]. Other mechanisms, such as the hydration theory, might be involved given the fact that MH could develop even after vitreous detachment [2]. A recent study reported that the volume reduction and the velocity decrease of the choriocapillaris is correlated with IMH [3].

Metabolic support of the retina and retinal pigment epithelium (RPE) is a crucial function of the choroid. According to Spaide et al., there exists an inverse relationship between the age and the thickness of the central choroid [4]. Moreover, a decline in both the density and the diameter of the choriocapillaris, along with a reduction in the blood flow within the choroid, can be observed with advancing age [3]. Ding et al. reported that central choroidal thickness (CCT) drastically reduces especially after sixties [5]. It is worth pointing out that the incidence of IMH is also known to peak around 65 years of age [6]. Several studies have found changes of the choroid by optical coherence tomography (OCT) and pointed out it as a cause of MH [7]. Reibaldi et al. [8] and Zeng et al. [9] reported that the patients with IMH have a thinner choroid, indicating a potential correlation between decreased choroidal blood flow and IMH. Moreover, there was direct research about the association of a reduced choriocapillaris blood flow with IMH [3].

Since OCT was introduced into ophthalmic clinical practice, a remarkable amount of research has been done on IMH. Enhanced depth imaging (EDI) methods in OCT even enabled observation of the choroid in vivo [4]. Recently developed OCT angiography (OCTA) technique enables visualization of the ocular vasculature without injecting additional agents [10]. Those techniques allow layer-by-layer observation of the vasculature from retina to even choroid. Previous studies have reported the choroidal characteristics of IMH observed by OCTA, but there are some limitations mainly due to relatively short follow-up period [11–13].

Pars plana vitrectomy (PPV) is the most conventional way to treat full-thickness MH (FTMH) currently. The efficacy of combined ILM peeling in facilitating MH closure has been established [14]. Many factors have been suggested as potential predictors of surgical outcomes in MH. Several studies have investigated that the preoperative MH morphological indices, such as minimum hole diameter (MD) [15], hole form factor (HFF) [16,17], macular hole index (MHI) [18,19], tractional hole index (THI) [20], macular hole closure index (MHCI) [21], central retinal thickness (CRT: the mean retinal thickness within 1-mm circle centered on the fovea) [22], hole diameter ratio (HDR) [23], etc. may predict surgical outcomes of MH. However, these biomarkers are based on the preoperative retinal morphology, and usually meant to predict only the closure rates of MH as the anatomical prognosis.

In this study, we observed the prognosis after MH surgery until the visual outcome stabilized. We investigated the correlation of the final visual outcome with the preoperative choroidal features analyzed by OCT/OCTA. Moreover, we assumed central foveal thickness (CFT: the retinal thickness at the foveal center) as an anatomical outcome after MH closure, and also examined its relationship with the choroidal features.

## Materials and methods

### Study population

This is a retrospective observational study which was conducted in the Department of Ophthalmology and Visual Science at Seoul St. Mary’s Hospital, the Catholic University of Korea. This study follows the principles outlined in the Declaration of Helsinki. The Institutional Review Board (IRB) of the Catholic University of Korea granted approval for all protocols in this study (KC23RASI0095). Given the retrospective nature of the study and the anonymization of data, the IRB has granted an exemption from written informed consent procedures. Data between January 4th, 2021 and May 31st, 2023 were accessed for this study from February 17th, 2023 to February 16th, 2024 in accordance with IRB approval.

27 patients above stage II IMH who underwent PPV with ILM peeling and perfluoropropane (C_3_F_8_) gas tamponade in our clinic from 2021 January to 2022 October were recruited to participate in this study. Their medical records were reviewed in a retrospective way. We excluded patients with a history of intravitreal injection, scleral buckling, or vitrectomy. Early stage (I) IMH or traumatic MH were also excluded. Patients with a history of uveitis, any other retinal diseases, or anterior segment diseases that could impact the surgical outcomes were not enlisted. Moreover, we excluded flap technique assisted PPV cases for controlled comparison. The staging was conducted by the latest system proposed by the International Vitreomacular Traction Study Group (IVTSG) which is based on size of the hole and status of the vitreous: stage 1, vitreomacular traction (VMT); stage 2, ≤ 400 μm FTMH with VMT; stage 3, > 400 μm FTMH with VMT; stage 4, complete posterior vitreous detachment (PVD) regardless of hole size [24].

### Study protocol

During the initial visit, relevant demographic data, as well as medical and ophthalmologic history, were gathered for all subjects. Slit-lamp microscopy was used to examine the subjects, and a dilated fundus examination was also conducted. Axial length of the study eye was measured preoperatively using IOL-Master 700 (Carl Zeiss Meditec, Jena, Germany). Snellen best-corrected visual acuity (BCVA) measurement and OCT/OCTA imaging were performed around 5 p.m. the day before the operation by DRI Triton SS-OCT (Topcon, Tokyo, Japan) which scans in the speed of 100,000 A-scans/s with a 1050-nm wavelength light source. Postoperative follow-up was done at 1, 3 and 6 months after surgery with measurements of BCVA and OCT during the afternoon outpatient appointments (2–4:30 p.m.), so diurnal variability on CCP and CCT was controlled. Two experienced retinal specialists (Y-H.P. and G-H.K.) who were blinded to the clinical histories of subjects independently reviewed and analyzed all OCT/OCTA images.

### Surgical technique

Under general anesthesia, a standard 25-gauge 3-port PPV (Constellation device, Alcon, Fort Worth, TX, USA) was conducted by the same experienced surgeon (Y-H.P.). If necessary, phacoemulsification and posterior chamber intraocular lens (Artis PL E; Cristalens Industrie, Lannion, France) implantation were carried out prior to the vitrectomy. A complete vitrectomy was performed in each patient, ensuring the elevation and trimming of the posterior hyaloid until reaching the peripheral vitreous base. Indocyanine green (ICG) staining was done in every patient, so that ILM was peeled off by forceps within an area of at least 2-disc diameter surrounding the MH. In this study, flap technique to improve surgical outcomes in the repair of MH was not conducted. A comprehensive examination of the peripheral retina was performed, and argon laser barrier photocoagulation was carried out around any degenerative lesions, retinal tears, or holes that were identified. In the end, the vitreous cavity was filled with 14% C_3_F_8_ gas. Patients were required to maintain a prone position for a minimum of one week following the surgery.

### OCT/OCTA image analysis

For preoperative evaluation. OCT/OCTA images were obtained the day prior to the surgery. Postoperative OCT images were captured for the eyes with IMH at 1, 3 and 6 months after the surgery. Image qualities of all OCT scans were above 65 and OCTA scans above 50. Images with motion artifacts and/or stretch artifacts were also excluded. Mean image quality of OCT was 89.07, and OCTA was 64.43 in this study.

**Fig 1:**
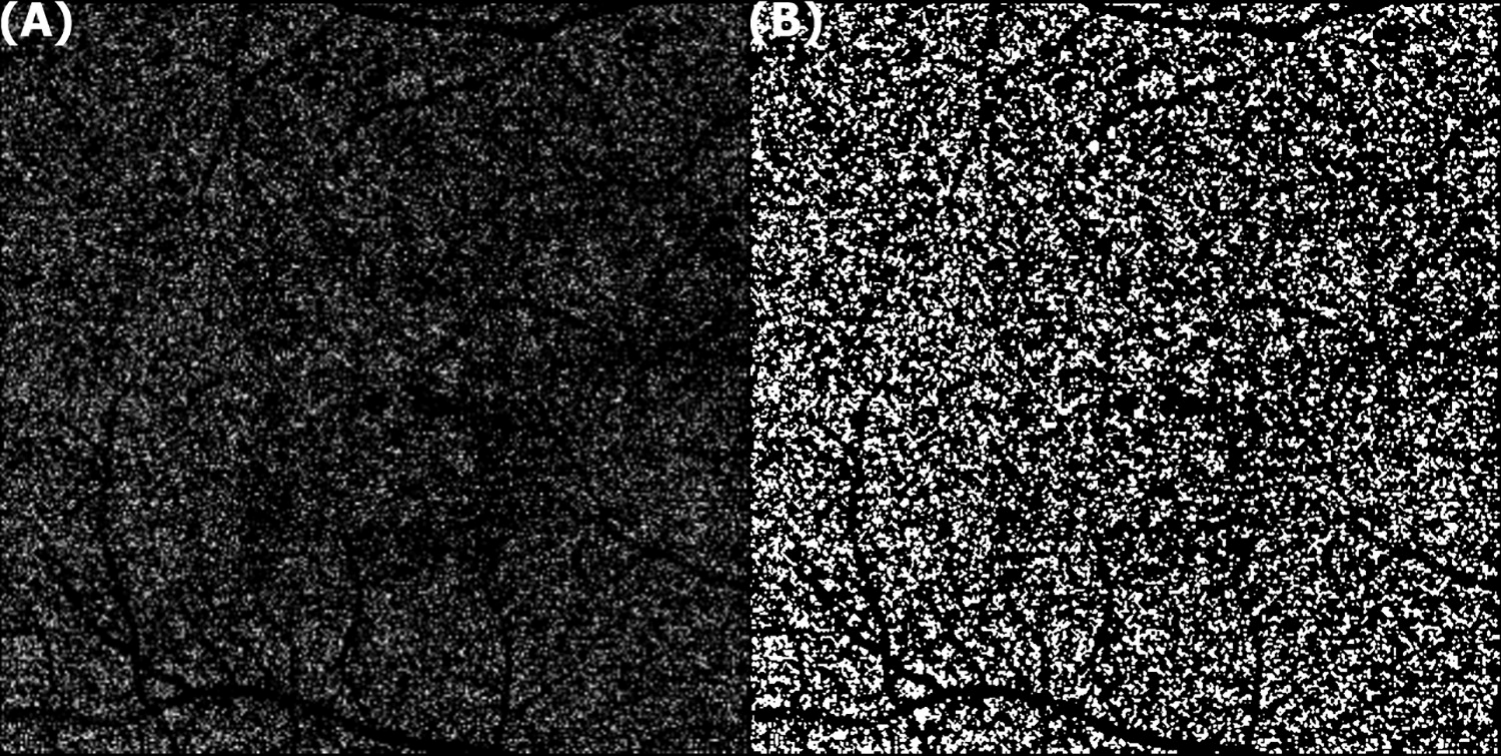
Choriocapillaris perfusion measurement with optical coherence tomography angiography (OCTA). (A) The en-face OCTA image of choriocapillaris was obtained from Topcon image-NET 6 software. (B) The image was binarized by Otsu’s autolocal threshold to demarcate the luminal and stromal area for measuring vascular densities by Image J software.

The 4.5 × 4.5 mm (320 × 320 pixels) en-face choriocapillaris images were automatically segmented by Topcon image-NET 6 software. The retinal projection artifacts were removed by the intrinsic projection artifact removal (PAR) tool before further image processing. Obtained images were converted into 8-bit images applied Otsu’s auto-local threshold for binarization to demarcate the luminal area and the stromal area as suggested by Nicolò et al. [25] These modified images were finally analyzed by the ‘Vascular Density (VD)’ command in Image J software (version 1.53a; National Institute of Health, USA) to achieve CCP (Fig 1).

**Fig 2:**
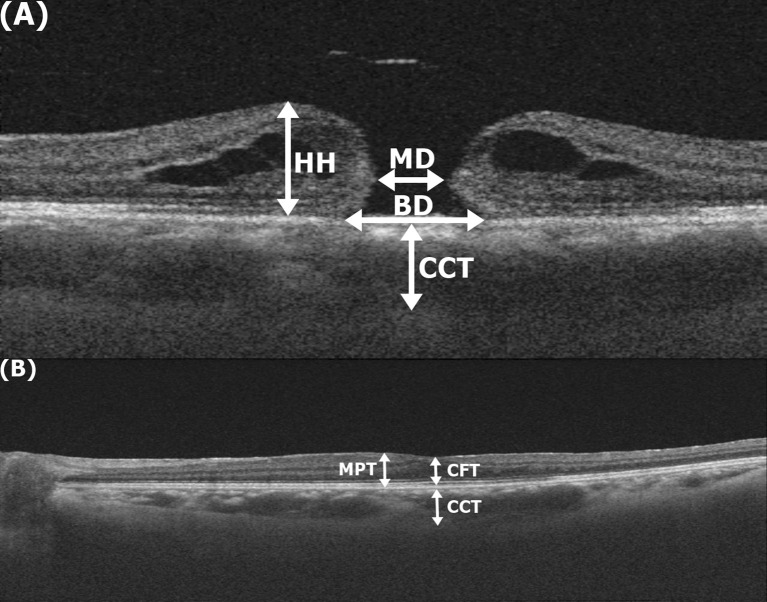
Morphological measurements with optical coherence tomography (OCT) using intrinsic digital caliper function. (A) Representative dimensions of macular hole were minimum/base diameter (MD/BD), hole height (HH) and central choroidal thickness (CCT) (B) Macular regeneration after surgery was assessed as central foveal thickness (CFT) and maximum parafoveal thickness (MPT).

We analyzed chorioretinal morphology using OCT images of the 12 mm x 12 mm 3D scan mode. Two experienced retinal specialists independently conducted measurements using the digital caliper tool available in Topcon image-NET 6 software, and they were averaged. Representative morphological features of MH were minimum diameter (MD), base diameter (BD), and hole height (HH). MD was measured at the nearest ends of the broken retinal tissue, while BD at the widest ends at the base. HH was defined as the maximum parafovea thickness of retinal tissue around MH. Postoperative regeneration of defected fovea was represented by central foveal thickness (CFT) and maximum parafoveal thickness (MPT). Central choroidal thickness (CCT) was measured before and after surgery as the vertical span between the hyper-reflective Bruch’s membrane line and the hyper-reflective inner surface of the sclera at fovea (Fig 2).

### Statistical analysis

Continuous data were expressed as the mean ± standard deviation (SD). Measured BCVA were transformed into the logarithm of the minimum angle of resolution (LogMAR). Prior to selecting the statistical analysis methods, the data’s normal distributions were assessed. Differences in BCVA, CCP, CCT, CFT and MPT were analyzed by Wilcoxon signed-rank test and Friedman test. Pearson correlation analysis identified correlations of preoperative parameters with functional and anatomical prognosis. Ultimately, all-in multiple linear regression analysis was performed to reveal significant biomarkers for postoperative outcomes. The statistical analysis was performed using the Statistical Package for the Social Sciences for Windows (Version 26.0; SPSS, Inc., Chicago, IL). *P*-values less than 0.05 were regarded as statistically significant.

## Results

Demographics and preoperative ocular characteristics are provided in Table 1. Data from 27 eyes of 27 patients (10 males and 17 females) with an age range from 57 to 72 years (mean 63.259 ± 6.975 years) were included in the study. Two eye (7.407%) were pseudophakic. Other 25 eyes (92.593%) had a clear phakic lens and underwent phacovitrectomy. There were 6 eyes at stage 2, 6 at stage 3, 15 at stage 4, and none at stage 1. Preoperative CCP and CCT of the fellow eyes were significantly lower than the study eyes (*p* < 0.001, *p* < 0.001). Measurements of MD, BD, HH, CCT, CFT and MPT by two researchers showed intraclass correlation coefficients of 0.985 (95% CI 0.971–0.993), 0.987 (95% CI 0.973–0.994), 0.982 (95% CI 0.968–0.990), 0.991 (95% CI 0.977–0.995), 0.989 (95% CI 0.972–0.994) and 0.990 (95% CI 0.974–0.995).

**Table 1:** Demographics and preoperative ocular characteristics.

Total number of eyes	27
Age (years)	63.259 ± 6.975
Gender (Male: Female)	10: 17
Laterality (Right: Left)	14: 13
Lens status (Phakia: Pseudophakia)	25: 2
Stage (1: 2: 3: 4)	0: 6: 6: 15
Minimum Diameter (MD) (μm)	334.852 ± 85.238
Base Diameter (BD) (μm)	590.370 ± 192.038
Hole Height (HH) (μm)	422.000 ± 75.700
Choriocapillaris Perfusion (CCP) (%)	27.335 ± 1.188
Central Choroidal Thickness (CCT) (μm)	256.074 ± 79.405
Axial Length (mm)	24.336 ± 1.794
Fellow eye’s CCP (%)	24.150 ± 1.295
Fellow eye’s CCT (μm)	220.222 ± 71.538

Data are presented as the mean ± standard deviation or a ratio, as appropriate.

**Fig 3:**
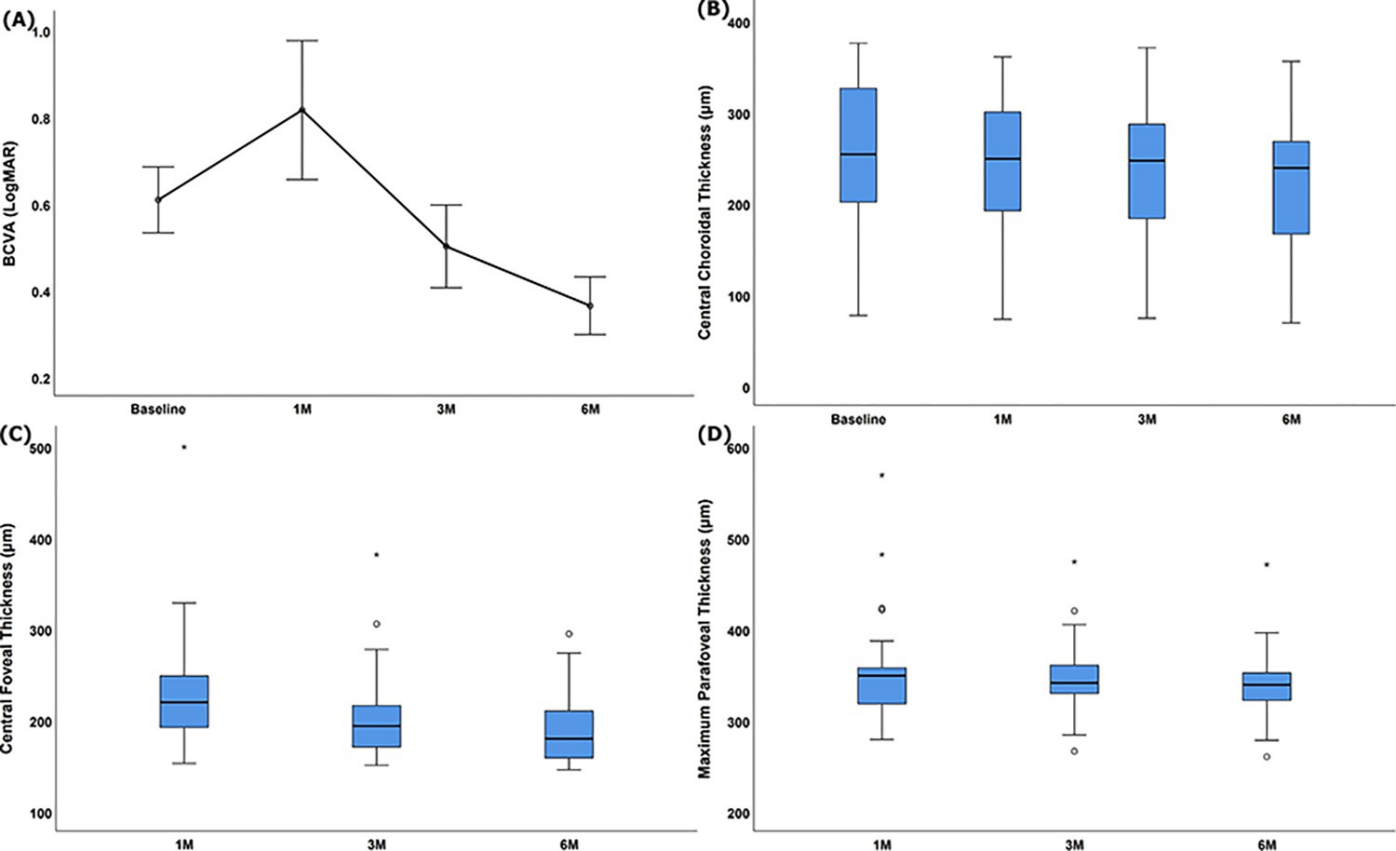
Changes in (A) best-corrected visual acuity (BCVA) (LogMAR, Logarithm of the minimum angle of resolution), (B) central choroidal thickness (CCT), (C) central foveal thickness (CFT) and (D) maximum parafoveal thickness (MPT) after macular hole repair.

As shown in Table 2, BCVA significantly improved from 3 months after the surgery (Fig 3A). Postoperative thinning of the choroid was revealed by comparison with the preoperative CCT (Fig 3B), and no significant difference was observed between postoperative CCT of the study eyes and the fellow eyes from 3 months after the surgery (1M, *p* = 0.031; 3M, *p* = 0.556; 6M, *p* = 0.220).

**Table 2:** Comparisons between the baseline and postoperative findings.

	Baseline	Postoperative			Friedman test (*P*-value)	Wilcoxon signed-rank test (*P*-value)					
		1M	3M	6M		Base vs. 1m	Base vs. 3m	Base vs. 6m	1m vs. 3m	1m vs. 6m	3m vs. 6m
BCVA (LogMAR)	0.607 ± 0.192	0.815 ± 0.405	0.500 ± 0.242	0.363 ± 0.169	**< 0.001**	0.082	**0.023**	**< 0.001**	**< 0.001**	**< 0.001**	**0.013**
CFT (μm)	n/a	231.333 ± 71.517	205.000 ± 71.517	189.222 ± 38.415	**< 0.001**	n/a	n/a	n/a	**< 0.001**	**< 0.001**	**0.006**
MPT (μm)	n/a	354.630 ± 60.452	345.556 ± 42.821	339.519 ± 39.926	**0.034**	n/a	n/a	n/a	**0.134**	**0.010**	0.276
CCT (μm)	256.074 ± 79.405	239.889 ± 77.362	230.852 ± 77.714	218.630 ± 74.163	**< 0.001**	**0.008**	**< 0.001**	**< 0.001**	0.058	**< 0.001**	**0.002**

Data are presented as the mean ± standard deviation.

Statistically significant *P*-value is shown in bold.

BCVA = Best-corrected visual acuity (LogMAR, Logarithm of the minimum angle of resolution); CFT = Central foveal thickness; MPT = Maximum parafoveal thickness; CCT = Central choroidal thickness.

**Fig 4:**
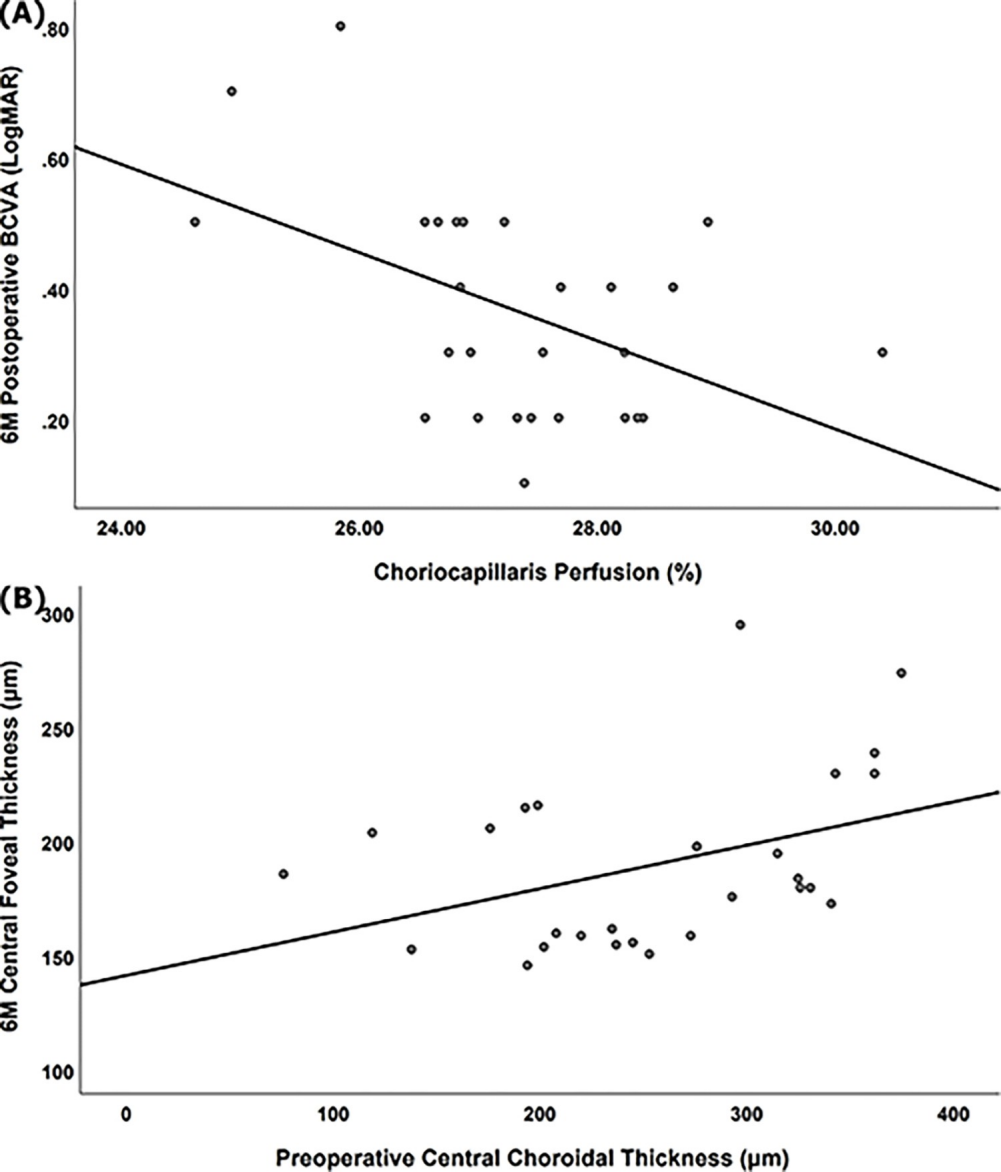
Scatterplots to exhibit correlations of (A) preoperative choriocapillaris perfusion (CCP) versus 6 months postoperative best-corrected visual acuity (BCVA) (LogMAR, Logarithm of the minimum angle of resolution) (R = 0.475, *p* = 0.012) and (B) preoperative central choroidal thickness (CCT) versus 6 months central foveal thickness (CFT) (R = 0.392, *p* = 0.043).

CFT and MPT also continued to reduce as time passes after surgery (Fig 3C and 3D). To figure out factors affecting surgical prognosis, preoperative BCVA, CCP, CCT, MD, BD, HH, axial length and conventional MH morphological indexes were evaluated using Pearson correlation analysis (Table 3). Final postoperative LogMAR BCVA was significantly associated positively with preoperative LogMAR BCVA (*p* = 0.007), MD (*p* = 0.031), BD (*p* = 0.026) and negatively with CCP (*p* = 0.012) (Fig 4A). CFT after 6 months from surgery, as an anatomical prognosis, was significantly related to preoperative CCT positively (*p* = 0.043) (Fig 4B). Age and axial length were not significantly related with CCP and CCT before and after surgery in our study.However, up-to-date MH morphological indexes (MHI, THI and HDR) were not definitely correlated to surgical outcomes in our study.

**Table 3:** Pearson correlation analysis between preoperative characteristics and final postoperative outcomes.

Preoperative	Postoperative BCVA		BCVA improvement		CFT		MPT	
	R	*p*	R	*p*	R	*p*	R	*p*
BCVA	**0.506**	**0.007**	**-0.590**	**0.001**	-0.084	0.676	-0.156	0.437
MD	**0.415**	**0.031**	0.059	0.771	-0.109	0.588	-0.163	0.415
BD	**0.429**	**0.026**	0.121	0.549	0.244	0.220	-0.117	0.559
HH	0.280	0.157	0.246	0.215	0.053	0.793	0.125	0.536
CCT	0.133	0.508	0.129	0.521	**0.392**	**0.043**	0.226	0.257
CCP	**-0.475**	**0.012**	0.019	0.924	-0.047	0.814	-0.091	0.651
MHI	-0.321	0.102	-0.115	0.568	-0.297	0.132	0.273	0.168
THI	-0.262	0.186	-0.036	0.857	0.147	0.463	0.197	0.325
HDR	-0.110	0.586	-0.085	0.675	-0.360	0.065	0.105	0.603
Axial length	-0.046	0.820	0.079	0.696	0.146	0.467	-0.352	0.072

Statistically significant *P*-value is shown in bold.

BCVA = Best-corrected visual acuity; MD = Minimum diameter; BD = Base diameter; HH = Hole height; CCT = Central choroidal thickness; CCP = Choriocapillaris perfusion; MHI = Macular hole index; THI = Tractional Hole Index; HDR = Hole diameter ratio; CFT = central foveal thickness; MPT = Maximum parafoveal thickness.

R, Correlation coefficient; *p*, Significant value.

Since multi-collinearities were not shown, all potential prognostic OCTA findings were adjusted for multiple linear regression analysis (Table 4). Preoperative CCP was significantly correlated with postoperative LogMAR BCVA (β = -0.403, *p* = 0.049). In case of postoperative CFT, preoperative CCT showed lowest *p*-value (= 0.065), but it lost statistical significance in multiple linear regression analysis.

**Table 4:** Multiple linear regression analysis for functional and anatomical outcomes.

Preoperative	6M postoperative BCVA (R^2^ = 0.393)			6M postoperative CFT (R^2^ = 0.296)		
	β	SE	*p*	β	SE	*p*
Age	-0.024	0.005	0.903	-0.203	1.145	0.341
CCP	**-0.403**	**0.027**	**0.049**	0.191	7.005	0.750
CCT	-0.074	<0.001	*0*.*700*	0.361	0.090	0.065
MD	0.207	<0.001	0.319	-0.231	0.100	0.722
BD	0.208	<0.001	0.356	0.396	0.048	0.603
HH	0.068	<0.001	0.741	-0.150	0.119	0.636

Statistically significant *P*-value is shown in bold.

BCVA = Best-corrected visual acuity (LogMAR = Logarithm of the minimum angle of resolution); CFT = central foveal thickness; CCP = Choriocapillaris perfusion; CCT = Central choroidal thickness; MD = Minimum diameter; BD = Base diameter; HH = Hole height.

R, Correlation coefficient; β, Standard regression coefficient; SE, Standard error; *p*, Significant value.

## Discussion

We analyzed the records of 27 patients to predict the surgical prognosis above stage II IMHs after PPV with ILM peeling. We found BCVA of IMH eyes was significantly improved by surgery and continued to improve until 6 months from the operation date. We also assumed CFT as an anatomical prognosis of IMH surgery by referring to past literature which showed the positive correlation between postoperative CRT and visual outcome [26,27]. In this study, we especially focused on the choroid/choriocapillaris, which is not structurally distorted by retinal changes, but closely influenced as the nearest layer. Our investigation of preoperative choroidal features regarding surgical prognosis showed the significant correlations of preoperative CCP and CCT respectively with postoperative BCVA and CFT in IMH. Preoperative CCT and CCP were significantly higher in IMH eyes than the fellow eyes. After surgery, CCT of IMH eyes gradually thinned so that no significant difference was observed compared to fellow eyes after 3 months from the operation date.

The associations between preoperative OCT measurements and prognosis of IMH after surgical treatment were firstly investigated by Ip et al., and the research indicated that IMH with a diameter below 400 μm demonstrated a superior recovery rate [15]. After that, HFF (= Left + Right oblique lengths / BD) [16], MHCI (Lengths of the detached photoreceptor arms / BD) [21], CRT [22], HDR (= MD / Maximum diameter) [23] have been found to be related with closure rate of IMH following the surgical intervention. Moreover, MHI (= HH / BD) [18,19], HFF [17], and THI (= HH / MD) [20] have been suggested to be correlated with postoperative BCVA. Majority of previous studies focused on the hole morphology for prediction of the surgical outcomes. These attempts to find predictors from the hole morphology are understandable considering the distinctive anatomical characteristics of IMH, but on the other hand, it seems several uncertain predictors are conflicting. Interestingly, up-to-date MH morphological indices, such as MHI, THI and HDR, showed no significant correlation with the surgical outcomes in this study, unlike MD or BD.

Two primary mechanisms, anteroposterior and tangential vitreous traction, have been proposed for IMH, but the exact pathogenesis is still uncertain. To find a good alternative predictor, it might be helpful to focus on another possible pathogenesis. The choroid consists mainly of blood vessels that provide oxygen and nutrients to the outer retina through the choriocapillaris [28]. Numerous studies have found that the choroidal features are related with development and healing of IMH [3,7–9].

Recently, OCTA uncovered remarkable results about the choriocapillaris flow area of eyes with IMH. Teng et al. demonstrated that there was a significant increase in choriocapillaris flow area and density a month after vitrectomy for IMH, but these changes were not correlated with visual outcomes [11]. Hwang et al. reported postoperative choriocapillaris flow void was strongly correlated with prognosis of visual acuity in nearly recovered IMH [12]. More recently, Endo H et al. found a mutualistic symbiotic relationship between components of the IMH-damaged photoreceptor/RPE/Bruch’s membrane/choriocapillaris complex [13]. In summary, significant improvements of the photoreceptor integrity, BCVA as well as the choriocapillaris vasculature after PPV were found in IMH. Above studies have led to attempts to predict the surgical prognosis based on the preoperative condition of the choroid/choriocapillaris. The functional restoration after the closure of MH was known to be accomplished after 6 months from the operation date. Hence, we extended the duration of the follow-up period compared to the above studies and successfully investigated the role of the preoperative choroid/choriocapillaris characteristics as prognostic factors for IMH surgery. Adequate choroidal blood flow is believed to be associated with outer retinal recovery and enhanced vision [12].

According to the previous studies [3,7–9], preoperative CCP and CCT should be lower in both eyes of unilateral IMH patients compared to normal individuals. Meanwhile, we compared both eyes of unilateral IMH patients and found preoperative CCP and CCT of IMH eyes were significantly higher than the fellow unaffected eyes. Moreover, CCT of IMH eyes became thinner after surgery, and no significant difference between both eyes was observed after 3 months from the operation date.

The traction force, which could be relieved by surgery, might be a plausible explanation for above findings of our study. The traction-induced alterations in retinal blood flow might stimulate the choroidal vessels to compensate for the oxygen and nutrient demands [29]. Moreover, the disruption of the retinal layer due to traction may lead to the compensation for the increased oxygen and nutrient needs with elevated vascular endothelial growth factor (VEGF) [30]. After resolution of traction force by surgery, above effects would diminish so CCT of IMH eyes becomes thinner to the level of fellow eyes.

This study enrolled patients with a 6-month follow-up appointment, enabling the investigation of changes that occurred over a long period compared with previous studies. However, there are some limitations. First one is its retrospective nature. Second, it included a relatively small number of cases, because OCTA was recently introduced for the preoperative evaluation of IMH in our clinic. Third disadvantage is lack of postoperative OCTA analysis due to practical reasons, even though many previous studies have consistently elucidated postoperative OCTA findings of improvements in choriocapillaris vasculature in IMH.

Our study is the first to support the hypothesis that preoperative CCP and CCT can be biomarkers for postoperative BCVA and CFT of IMH surgery. Furthermore, it may provide evidence about pathogenesis and healing process of IMH. However, it is still unclear how choroidal flow contributes to the evolution of IMH and unable to assess the causal relationship of our findings in IMH. We hope that future research will prospectively confirm the significant results of our study. Additionally, verifying our findings in other clinics or populations would be beneficial for enhancing external validity in the future.

## Supporting information

S1 Table Raw data for replicating this study findings. (PDF)

S1 File Zip file containing en-face OCTA choriocapillaris images of enrolled eyes. (ZIP)
