# Supplementary material for: Analysis of choroidal features to predict surgical prognosis of idiopathic macular hole
Source: PLoS One. 2024 Sep 6;19(9):e0308292. doi: 10.1371/journal.pone.0308292 (PMC11379149; doi:10.1371/journal.pone.0308292)
Supplement: S1 Table — (PDF) [file pone.0308292.s001.pdf]

							Hole morphology			Choroidal features					LogMAR BCVA				Closed hole morphology						Fellow eye	
Pt. No.	Gender	Age	Cormobidity	Op. date	Laterality	AxL	BD	MD	HH	CCP	CCT_base	CCT_1m	CCT_3m	CCT_6m	VA_base	VA_1m	VA_3m	VA_6m	MPT_1m	MPT_3m	MPT_6m	CFT_1m	CFT_3m	CFT_6m	TOE CPD	TOE CCT
1	M	64	DM	2022.05.04	OS	25.77	660	270	363	27.332	362	355	344	337	0.6	0.3	0.2	0.2	352	340	321	250	237	229	22.735	359
2	F	52	HTN	2022.04.06	OS	23.58	570	300	438	26.939	375	360	370	355	0.6	1.2	0.3	0.3	351	347	344	280	277	273	24.161	313
3	F	56	none	2022.06.08	OD	24.12	846	225	450	26.853	297	265	251	251	0.5	1.2	0.4	0.4	386	377	355	312	305	294	28.989	292
4	M	72	none	2021.12.29	OS	23.33	856	355	540	26.756	325	280	270	267	0.5	0.5	0.2	0.3	315	325	326	246	205	183	23.964	247
5	M	69	DM	2021.06.08	OS	23.86	682	502	379	27.698	343	297	300	287	0.7	0.7	0.5	0.4	314	296	311	240	224	229	23.753	310
6	M	65	none	2021.07.13	OS	26.74	466	251	445	30.404	199	198	162	158	0.3	0.9	1	0.3	284	285	289	208	199	215	25.7	188
7	M	68	HTN	2021.12.01	OD	24.7	812	368	343	25.846	176	168	163	158	1	1	0.8	0.8	301	283	277	219	216	205	23.44	170
8	M	70	none	2022.01.26	OS	28.2	642	358	509	28.239	193	151	139	155	0.6	0.4	0.3	0.2	343	338	345	221	195	214	22.364	175
9	F	58	HTN	2021.06.16	OD	27.84	619	414	385	27.449	76	72	73	68	0.4	1.1	0.9	0.2	310	326	317	205	193	185	23.558	77
10	F	40	none	2021.02.10	OD	28.9	623	458	601	26.555	237	183	183	150	0.7	1	0.8	0.5	278	265	259	152	156	154	24.499	167
11	M	62	none	2022.09.13	OS	25.98	615	323	454	26.823	362	302	295	294	0.7	2	0.5	0.5	422	419	395	314	267	238	22.353	307
12	F	69	none	2021.01.19	OS	21.9	481	353	415	28.936	119	101	97	86	0.7	1	0.6	0.5	349	348	341	241	215	203	23.533	111
13	F	55	none	2022.08.23	OS	23.76	456	329	338	26.666	315	320	277	248	0.5	1.7	0.5	0.5	315	335	321	208	201	194	23.187	220
14	M	70	HTN	2021.06.30	OD	25.07	676	398	410	27.223	253	242	231	198	0.7	0.7	0.5	0.5	348	341	338	209	179	150	24.891	227
15	F	65	HTN	2021.02.16	OS	23.21	716	348	578	28.122	220	219	203	197	0.5	0.7	0.7	0.4	481	387	391	328	173	158	23.7	216
16	M	71	HTN	2021.09.28	OD	23.53	396	341	364	28.234	245	248	246	238	0.6	0.6	0.4	0.3	362	372	363	198	157	155	24.84	195
17	F	62	none	2021.10.19	OS	22.91	526	368	409	27.68	341	337	329	318	0.2	0.4	0.3	0.2	348	367	351	191	188	172	23.109	119
18	F	68	none	2022.10.25	OD	23.62	445	329	339	28.388	194	184	194	192	0.7	0.4	0.3	0.2	319	331	315	161	150	145	23.844	141
19	F	64	none	2021.10.26	OD	22.87	825	524	467	24.622	293	269	261	260	1	0.6	0.5	0.5	343	338	337	234	183	175	24.245	253
20	F	63	HTN	2021.01.05	OD	23.68	343	328	432	26.878	276	260	246	245	0.5	0.9	0.7	0.5	420	404	470	222	208	197	23.568	231
21	F	63	none	2021.07.16	OD	23.13	309	136	339	27.001	208	214	210	193	0.7	0.5	0.3	0.2	358	352	351	169	167	159	24.689	180
22	F	67	none	2021.05.11	OD	23.87	297	210	356	26.556	273	262	259	241	0.8	0.6	0.3	0.2	340	337	325	166	162	158	23.262	255
23	F	67	HTN	2022.07.19	OS	22.46	322	309	359	28.344	235	222	210	189	0.7	0.5	0.3	0.2	322	318	325	251	191	161	24.145	217
24	F	60	none	2021.03.05	OD	23.62	703	343	499	24.931	331	330	321	281	0.7	1	0.9	0.7	568	473	375	499	381	179	25.29	317
25	F	63	HTN	2021.01.05	OD	23.19	1034	379	483	28.642	202	199	182	173	0.8	1	0.7	0.4	343	339	351	160	155	153	24.375	213
26	F	57	HTN	2021.01.05	OS	22.85	664	297	364	27.547	326	320	315	267	0.4	0.8	0.5	0.3	348	345	340	192	188	179	24.406	301
27	M	68	HTN	2021.01.08	OD	24.39	356	225	335	27.391	138	119	102	97	0.3	0.3	0.1	0.1	355	342	334	170	163	152	25.437	145
