# Supplementary figures and images for: Analysis of choroidal features to predict surgical prognosis of idiopathic macular hole
Source: PLoS One. 2024 Sep 6;19(9):e0308292. doi: 10.1371/journal.pone.0308292 (PMC11379149; doi:10.1371/journal.pone.0308292)

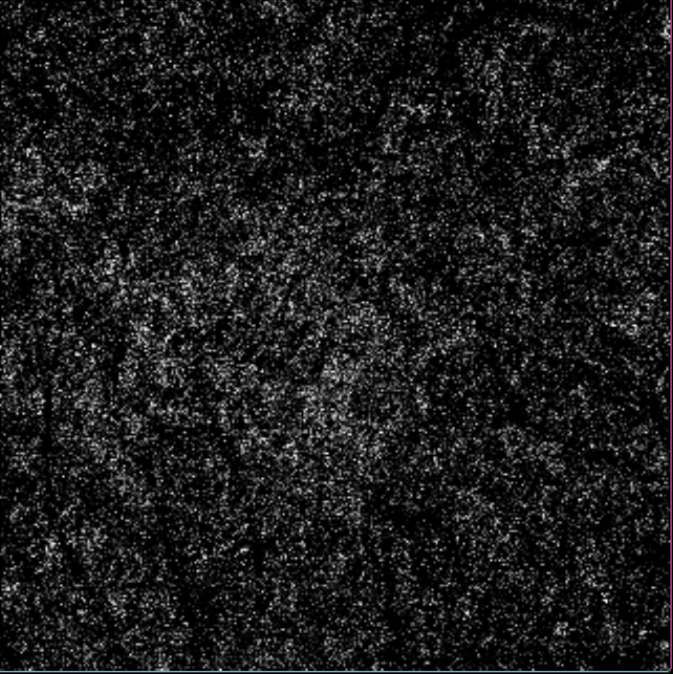

Supplement: S1 File — (ZIP) [file pone.0308292.s002.zip › Fellow eyes/1.PNG]

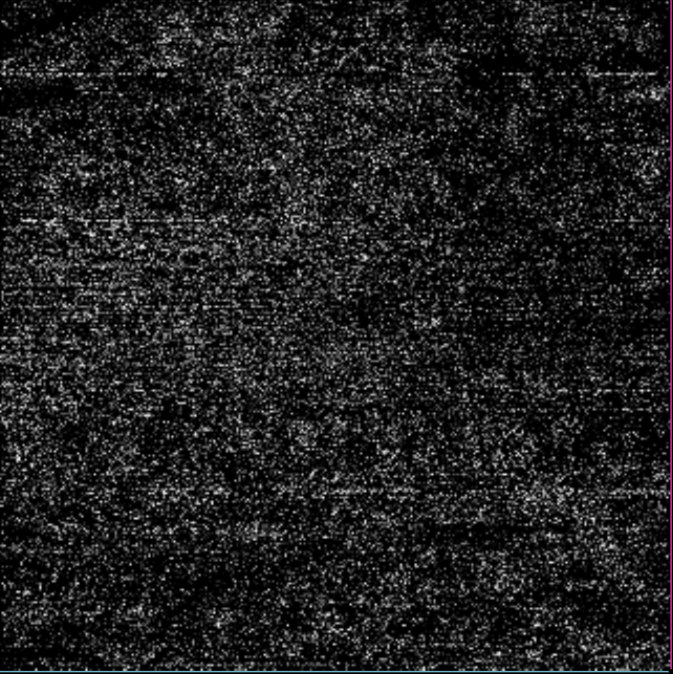

Supplement: S1 File — (ZIP) [file pone.0308292.s002.zip › Fellow eyes/10.PNG]

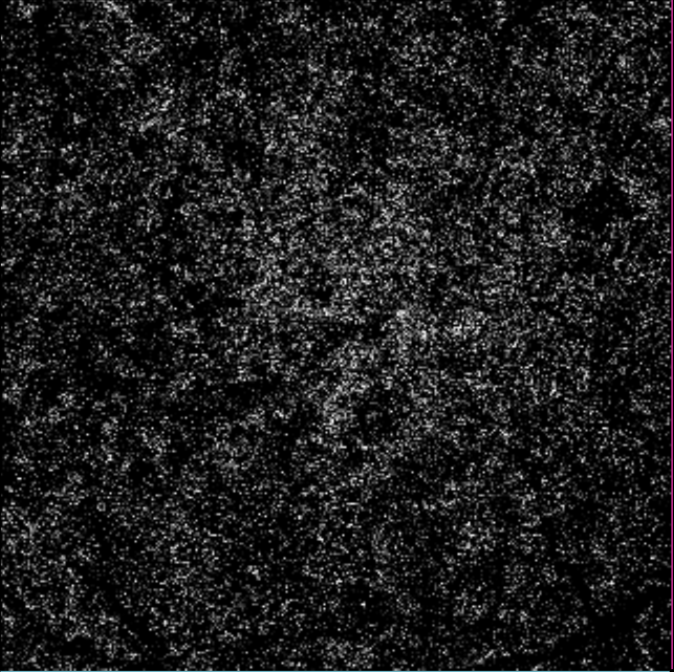

Supplement: S1 File — (ZIP) [file pone.0308292.s002.zip › Fellow eyes/11.PNG]

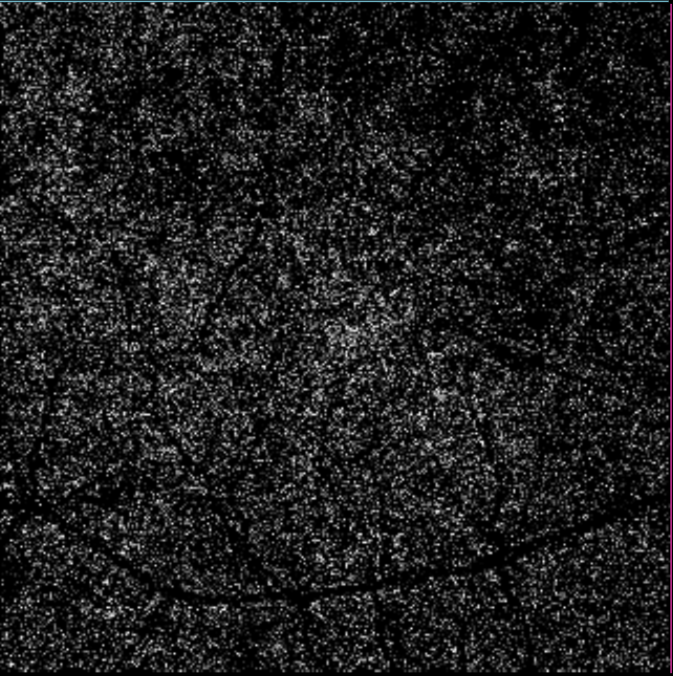

Supplement: S1 File — (ZIP) [file pone.0308292.s002.zip › Fellow eyes/12.PNG]

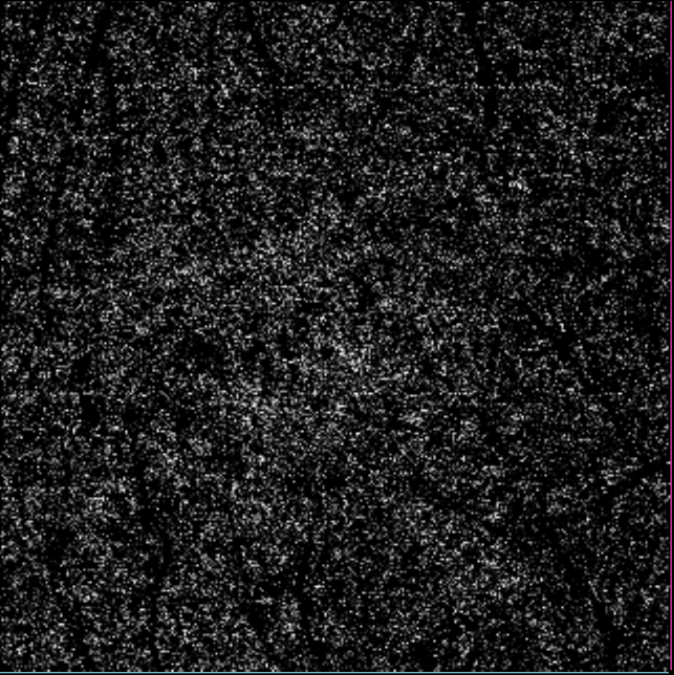

Supplement: S1 File — (ZIP) [file pone.0308292.s002.zip › Fellow eyes/13.PNG]

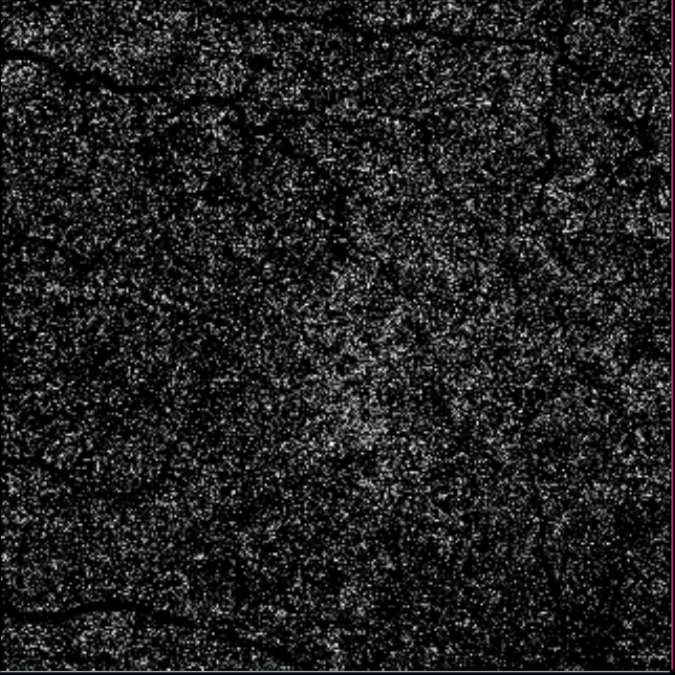

Supplement: S1 File — (ZIP) [file pone.0308292.s002.zip › Fellow eyes/14.PNG]

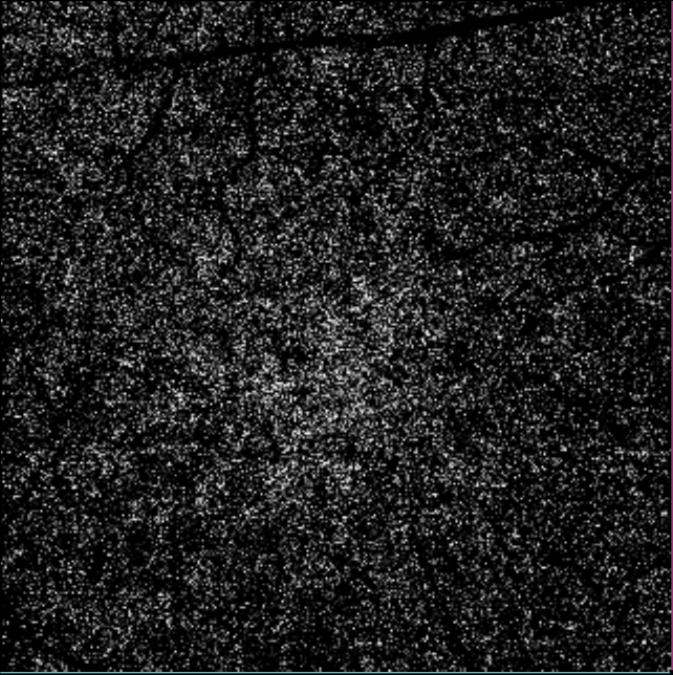

Supplement: S1 File — (ZIP) [file pone.0308292.s002.zip › Fellow eyes/15.PNG]

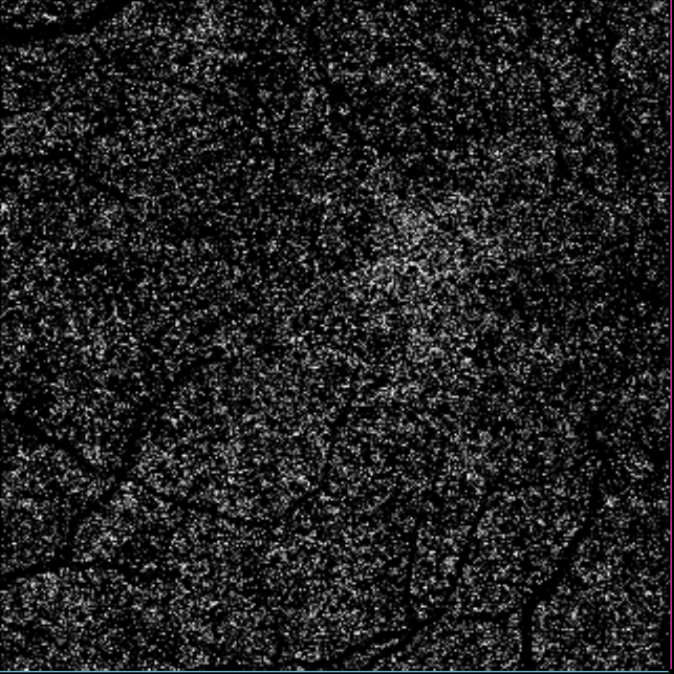

Supplement: S1 File — (ZIP) [file pone.0308292.s002.zip › Fellow eyes/16.PNG]

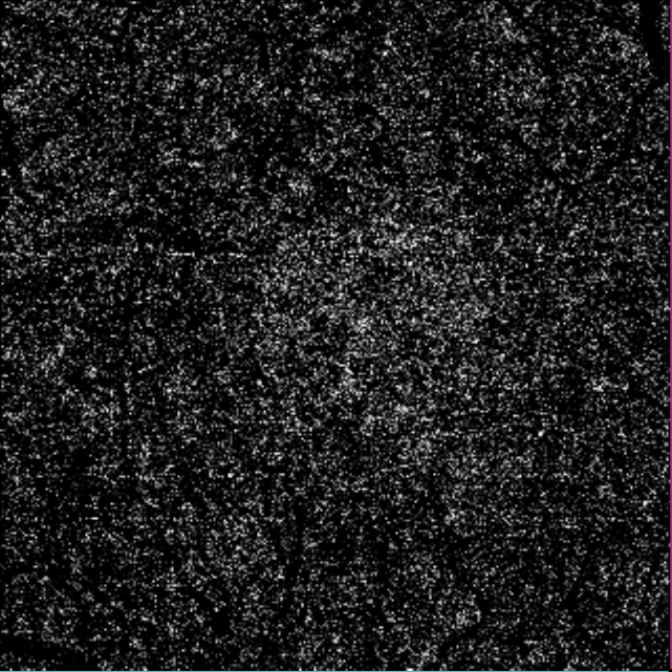

Supplement: S1 File — (ZIP) [file pone.0308292.s002.zip › Fellow eyes/17.PNG]

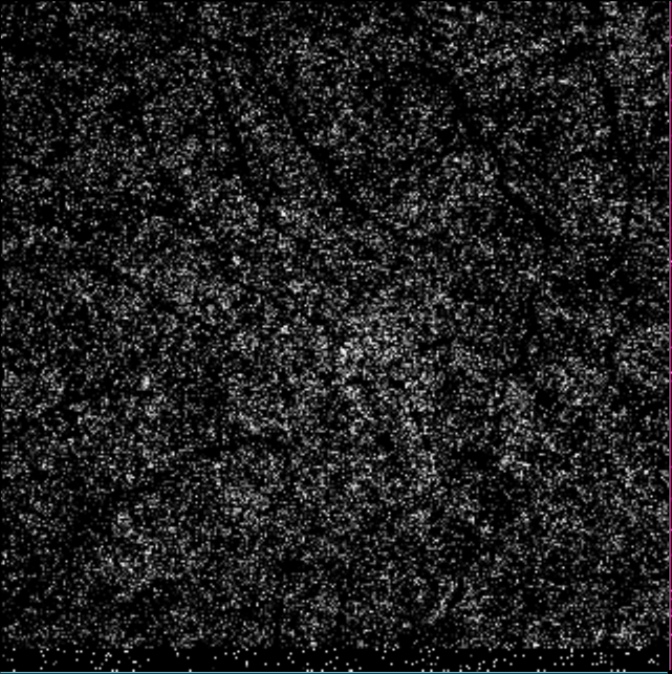

Supplement: S1 File — (ZIP) [file pone.0308292.s002.zip › Fellow eyes/18.PNG]

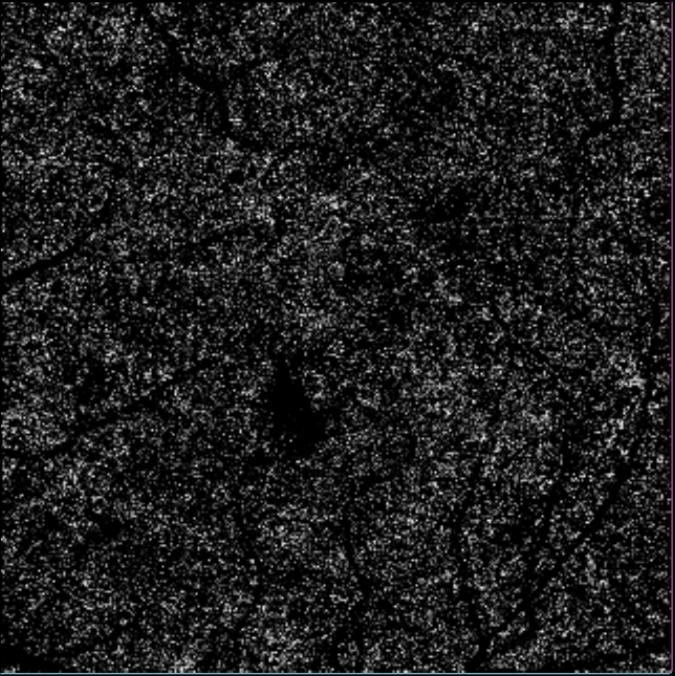

Supplement: S1 File — (ZIP) [file pone.0308292.s002.zip › Fellow eyes/19.PNG]

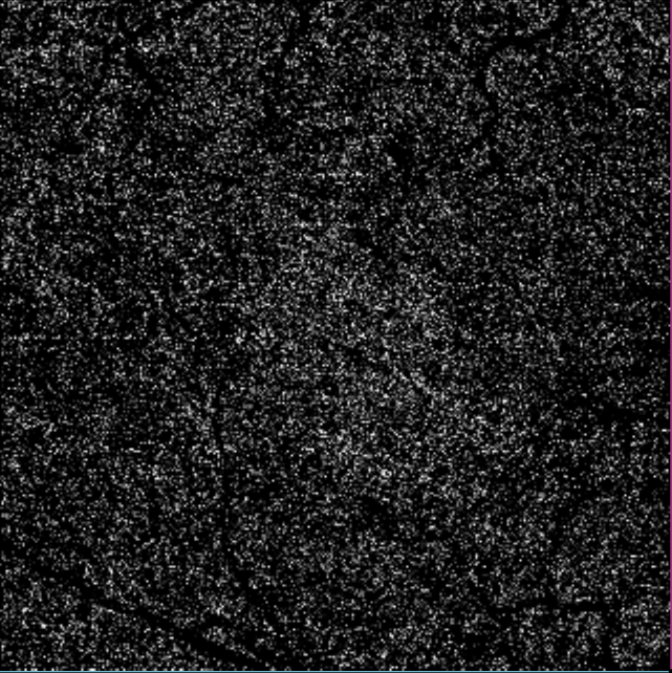

Supplement: S1 File — (ZIP) [file pone.0308292.s002.zip › Fellow eyes/2.PNG]

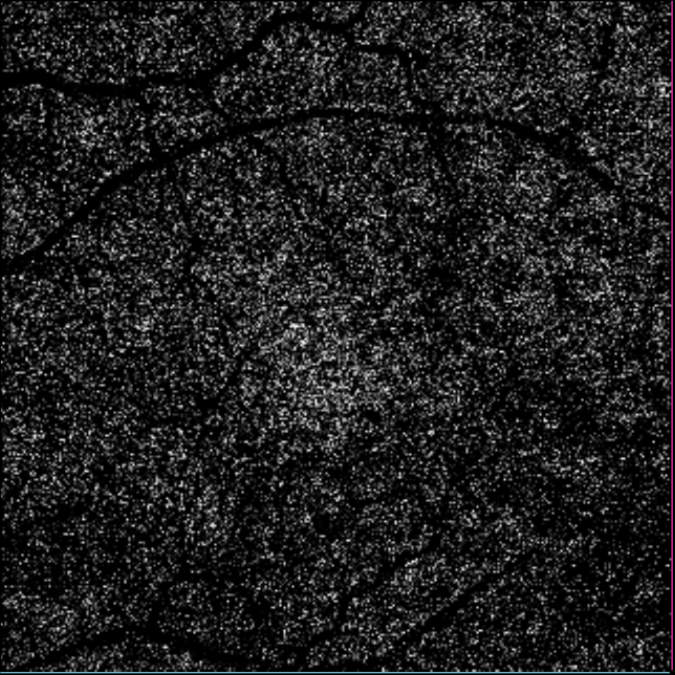

Supplement: S1 File — (ZIP) [file pone.0308292.s002.zip › Fellow eyes/20.PNG]

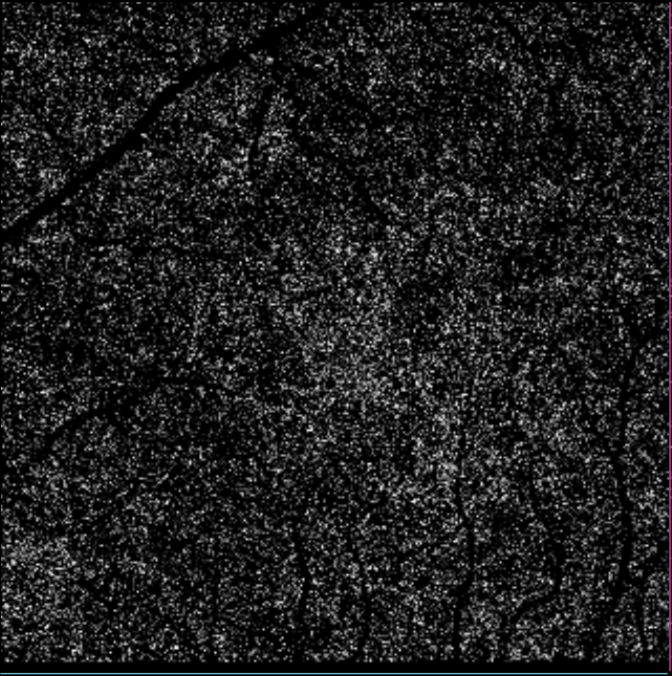

Supplement: S1 File — (ZIP) [file pone.0308292.s002.zip › Fellow eyes/21.PNG]

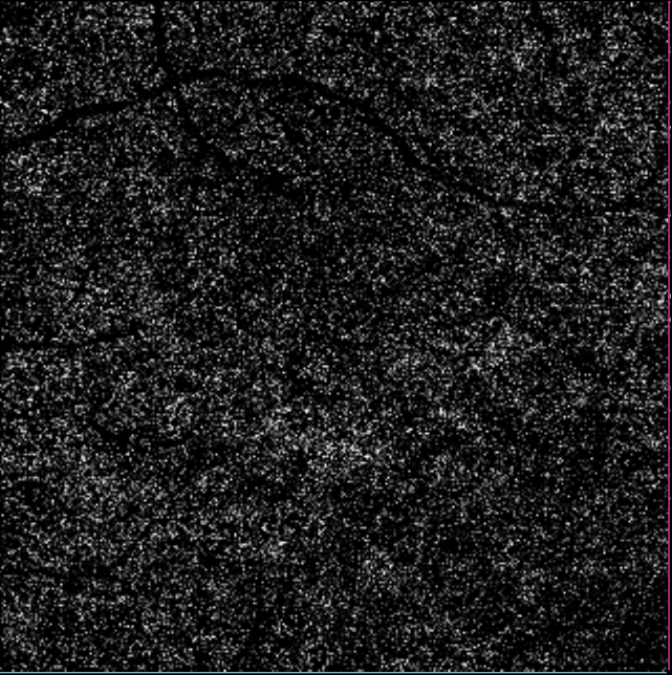

Supplement: S1 File — (ZIP) [file pone.0308292.s002.zip › Fellow eyes/22.PNG]

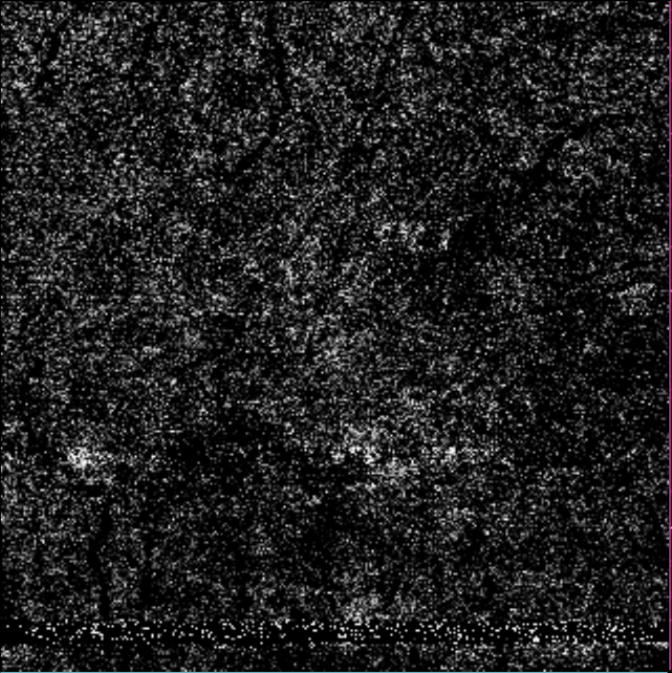

Supplement: S1 File — (ZIP) [file pone.0308292.s002.zip › Fellow eyes/23.PNG]

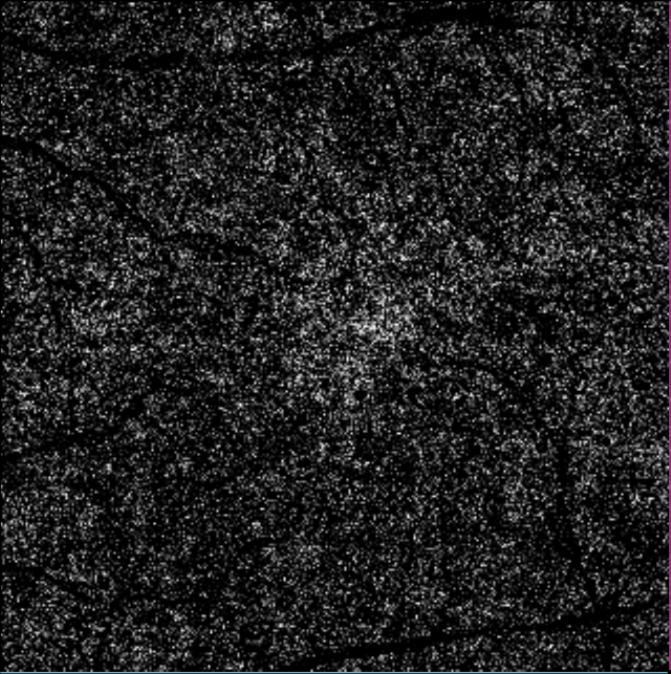

Supplement: S1 File — (ZIP) [file pone.0308292.s002.zip › Fellow eyes/24.PNG]

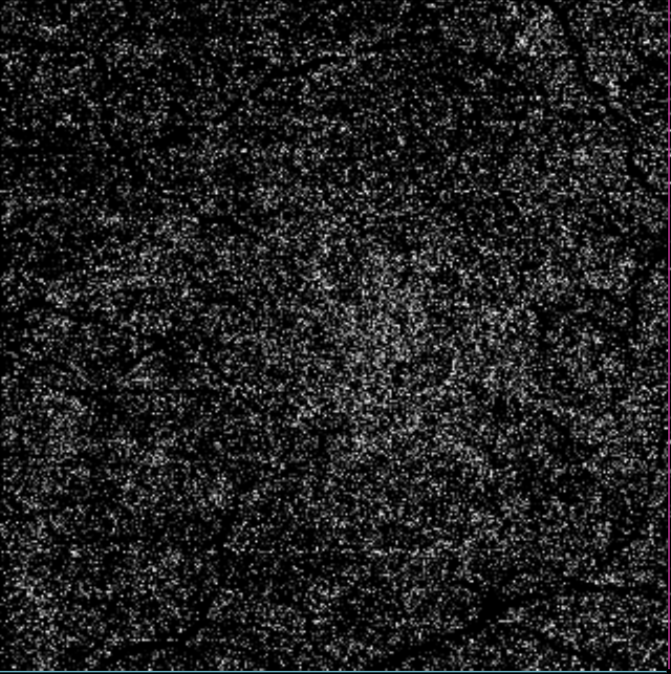

Supplement: S1 File — (ZIP) [file pone.0308292.s002.zip › Fellow eyes/25.PNG]

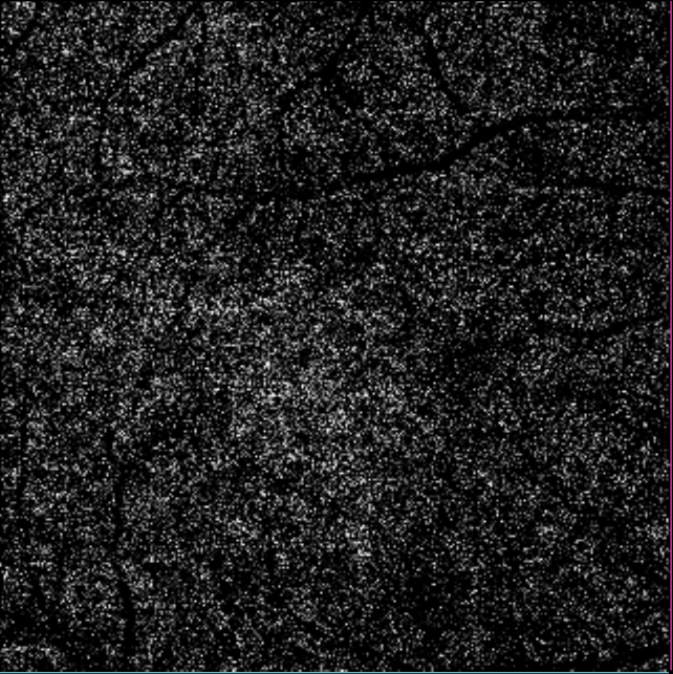

Supplement: S1 File — (ZIP) [file pone.0308292.s002.zip › Fellow eyes/26.PNG]

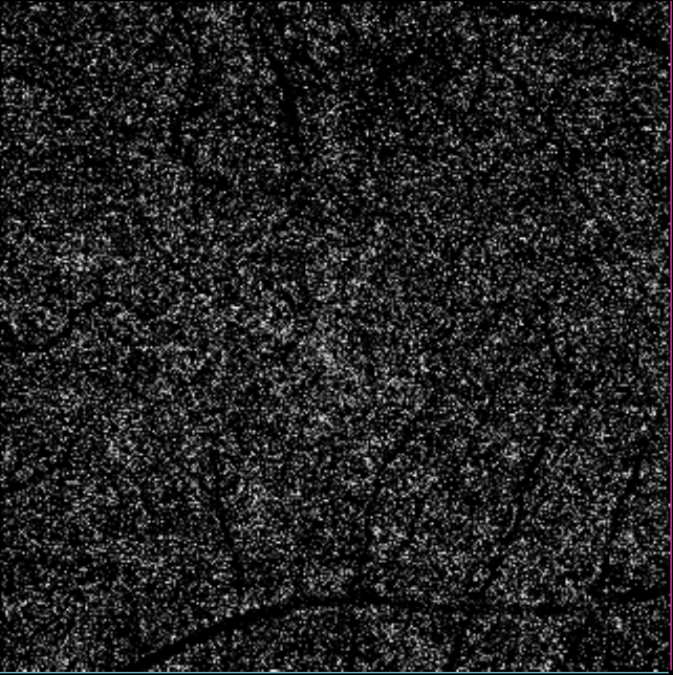

Supplement: S1 File — (ZIP) [file pone.0308292.s002.zip › Fellow eyes/27.PNG]

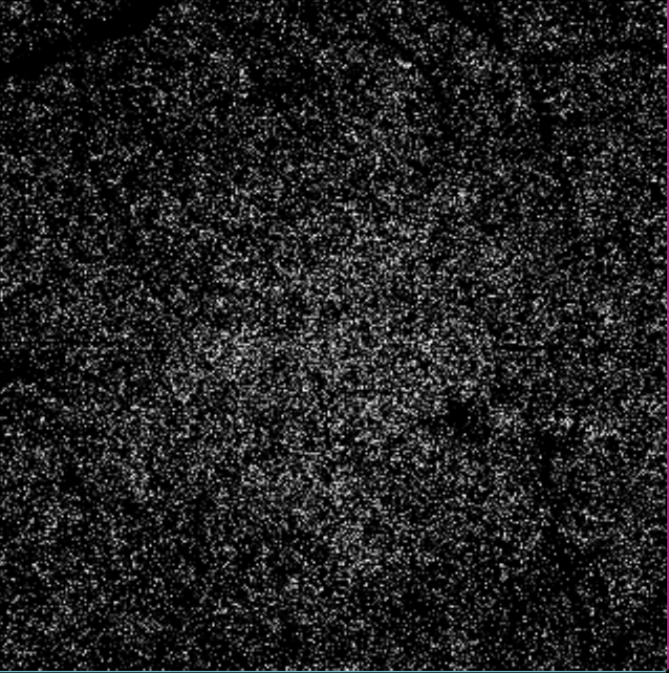

Supplement: S1 File — (ZIP) [file pone.0308292.s002.zip › Fellow eyes/3.PNG]

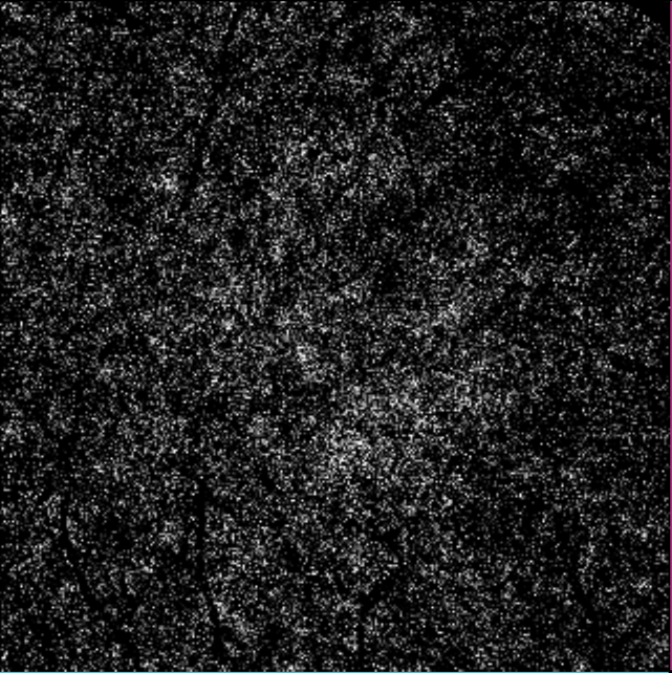

Supplement: S1 File — (ZIP) [file pone.0308292.s002.zip › Fellow eyes/4.PNG]

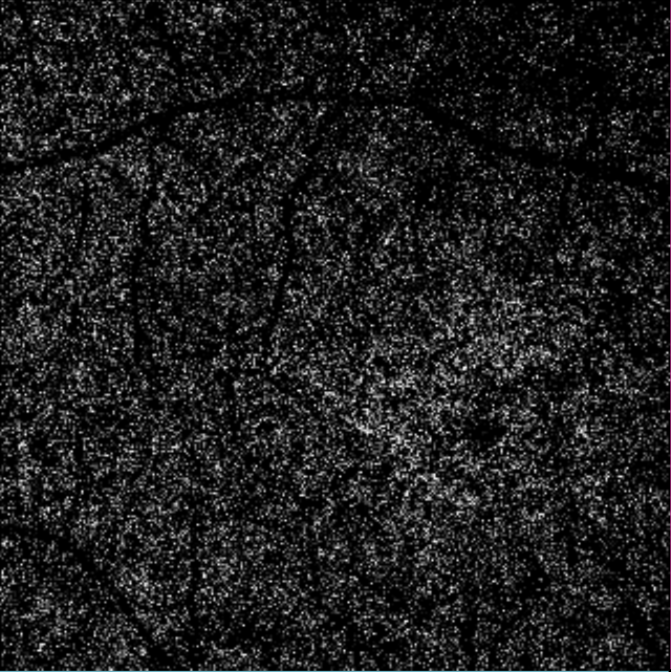

Supplement: S1 File — (ZIP) [file pone.0308292.s002.zip › Fellow eyes/5.PNG]

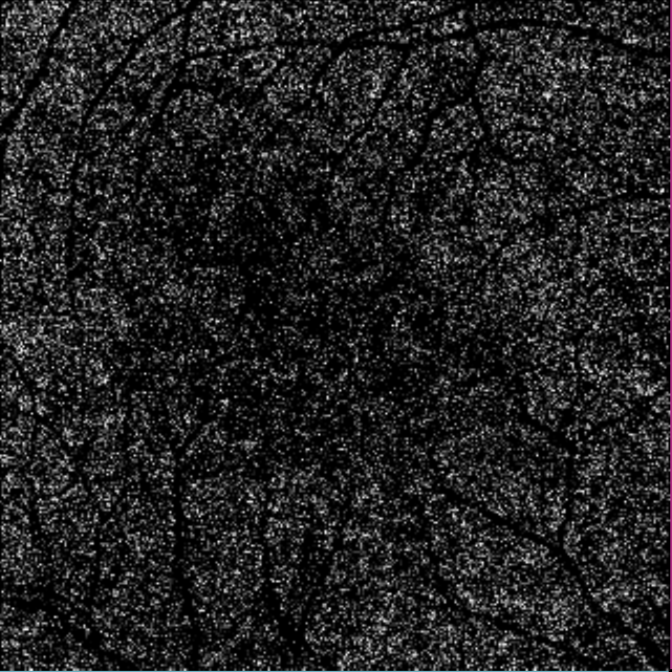

Supplement: S1 File — (ZIP) [file pone.0308292.s002.zip › Fellow eyes/6.PNG]

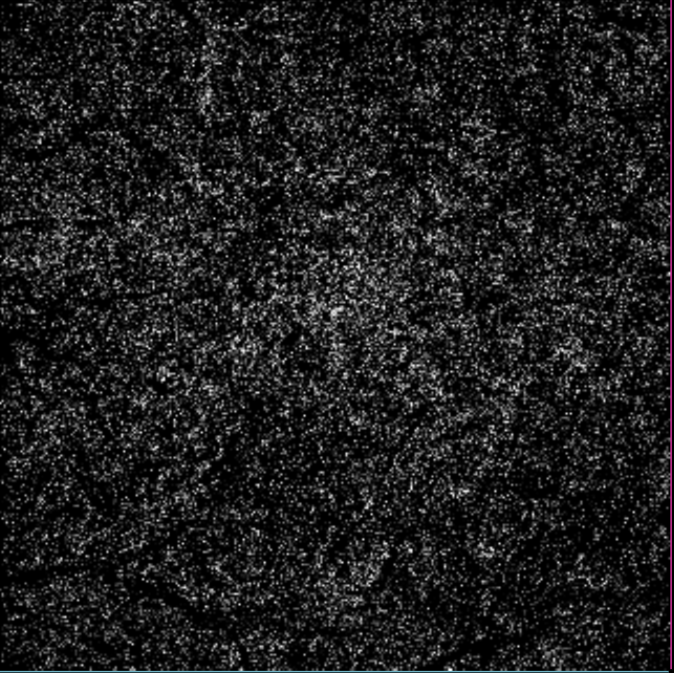

Supplement: S1 File — (ZIP) [file pone.0308292.s002.zip › Fellow eyes/7.PNG]

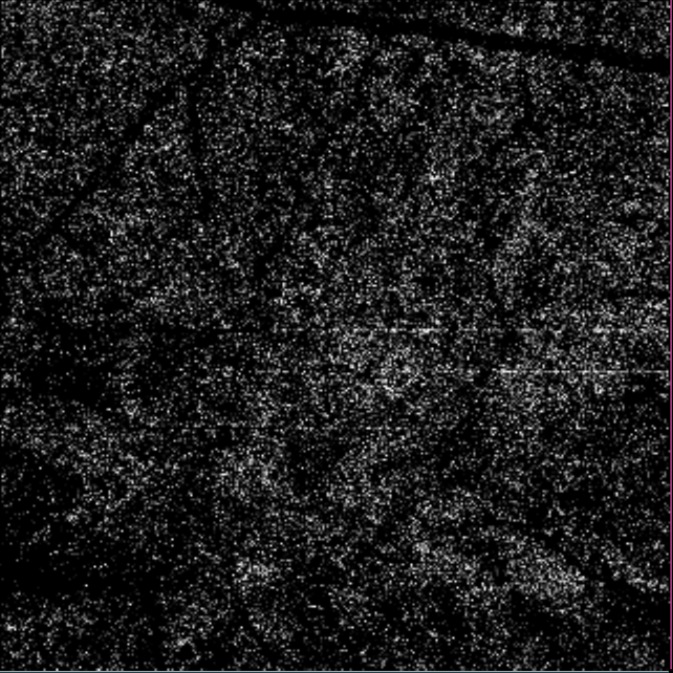

Supplement: S1 File — (ZIP) [file pone.0308292.s002.zip › Fellow eyes/8.PNG]

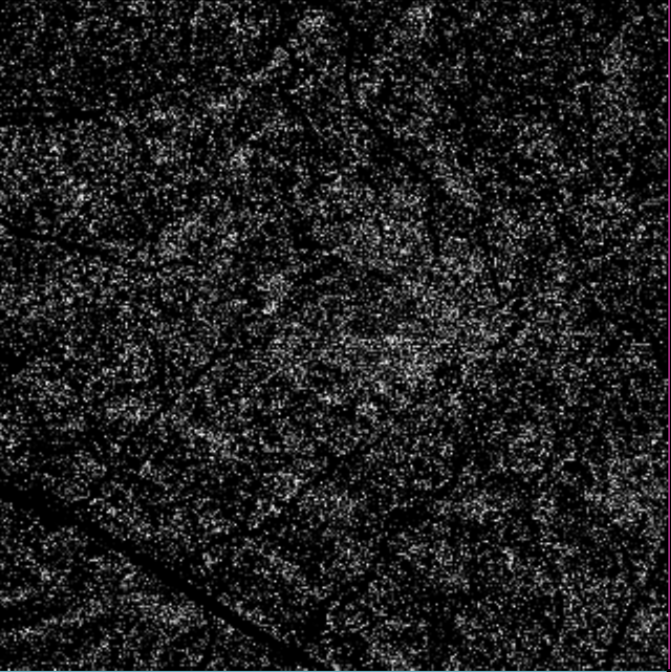

Supplement: S1 File — (ZIP) [file pone.0308292.s002.zip › Fellow eyes/9.PNG]

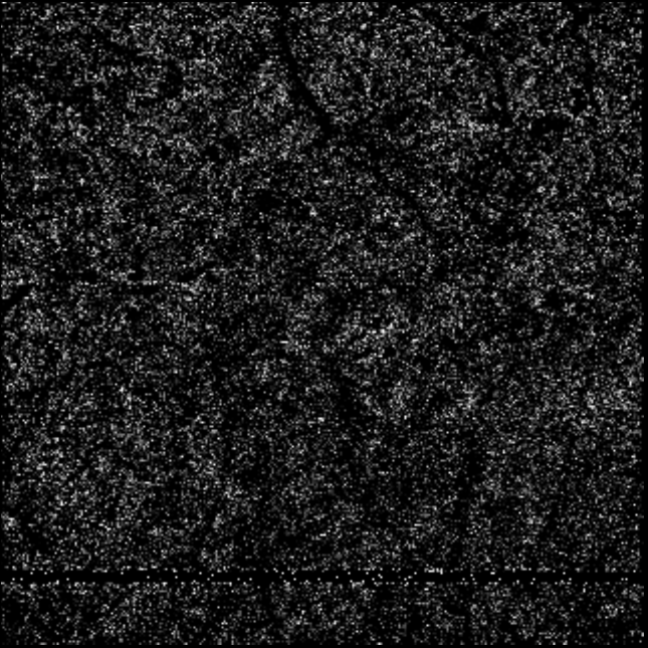

Supplement: S1 File — (ZIP) [file pone.0308292.s002.zip › Study eye/1.png]

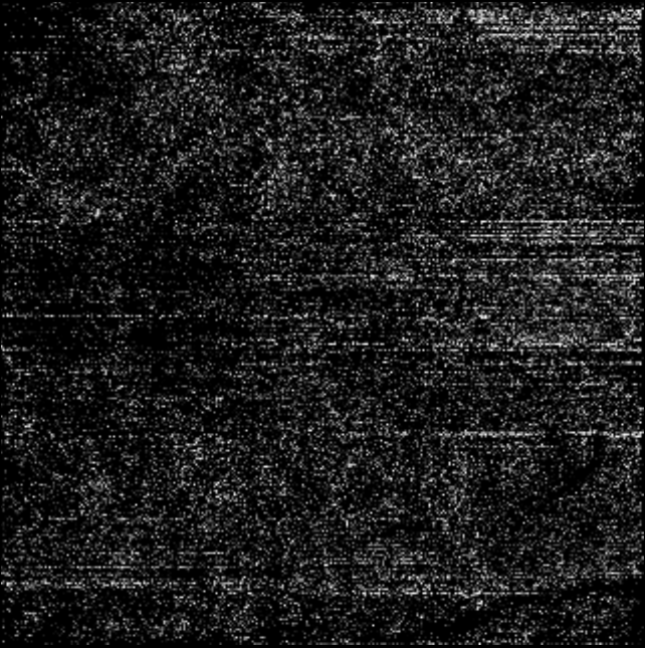

Supplement: S1 File — (ZIP) [file pone.0308292.s002.zip › Study eye/10.png]

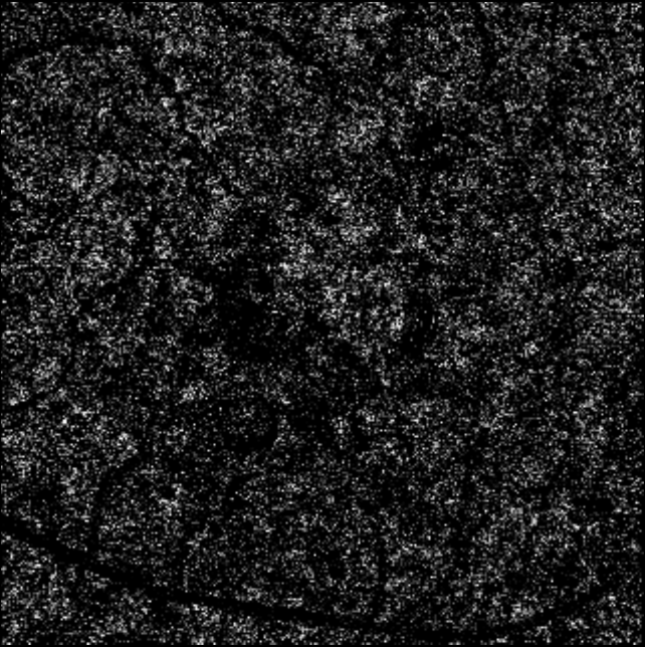

Supplement: S1 File — (ZIP) [file pone.0308292.s002.zip › Study eye/11.png]

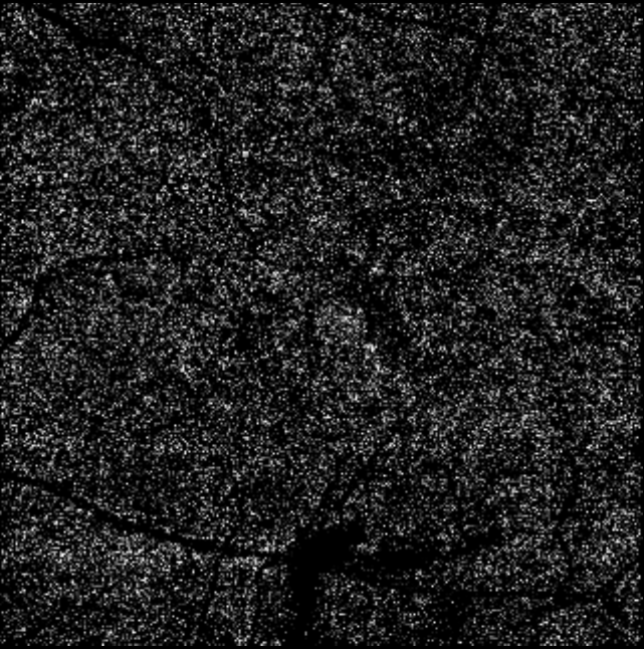

Supplement: S1 File — (ZIP) [file pone.0308292.s002.zip › Study eye/12.png]

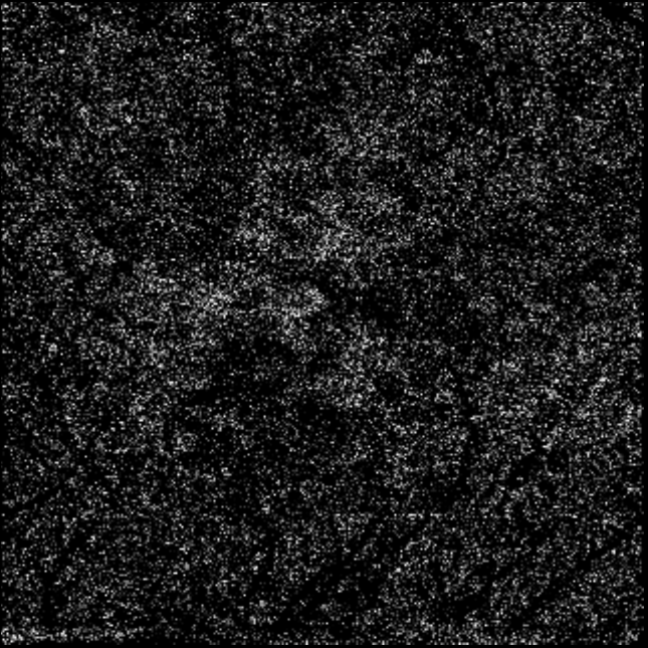

Supplement: S1 File — (ZIP) [file pone.0308292.s002.zip › Study eye/13.png]

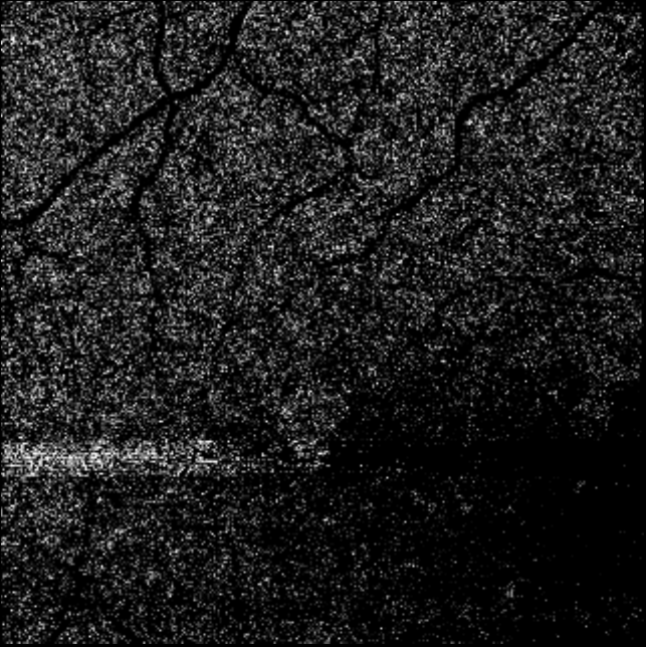

Supplement: S1 File — (ZIP) [file pone.0308292.s002.zip › Study eye/14.png]

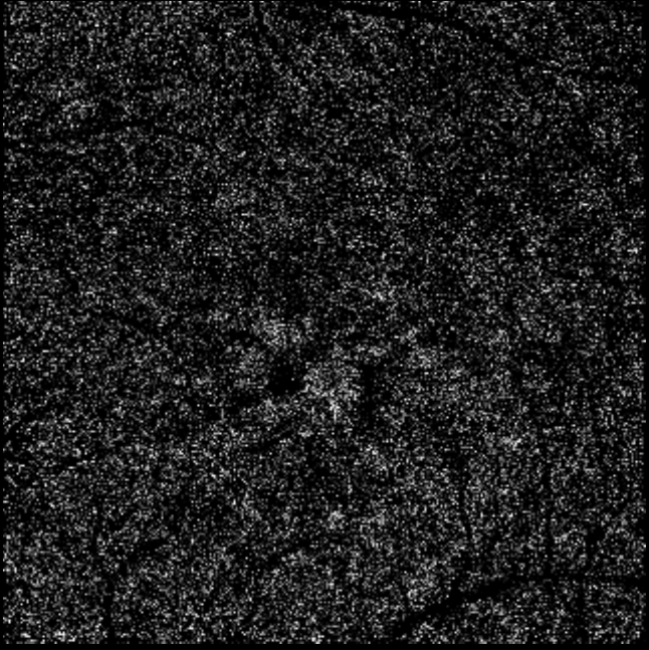

Supplement: S1 File — (ZIP) [file pone.0308292.s002.zip › Study eye/15.png]

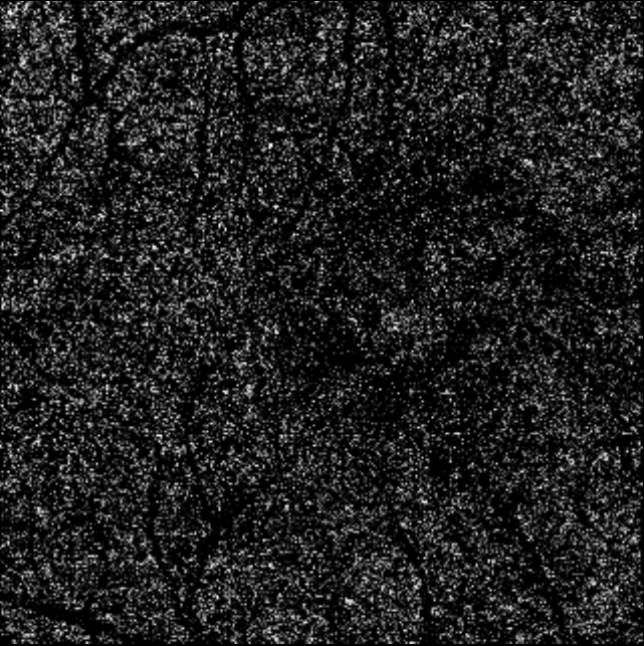

Supplement: S1 File — (ZIP) [file pone.0308292.s002.zip › Study eye/16.png]

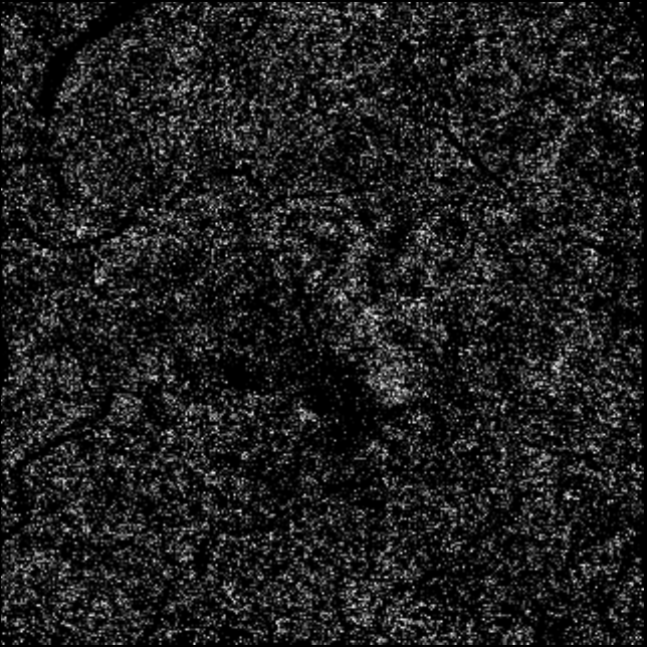

Supplement: S1 File — (ZIP) [file pone.0308292.s002.zip › Study eye/17.png]

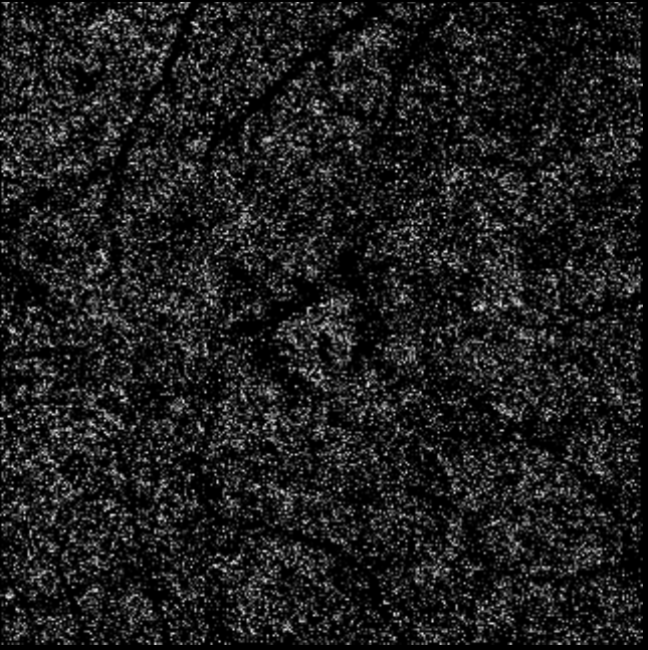

Supplement: S1 File — (ZIP) [file pone.0308292.s002.zip › Study eye/18.png]

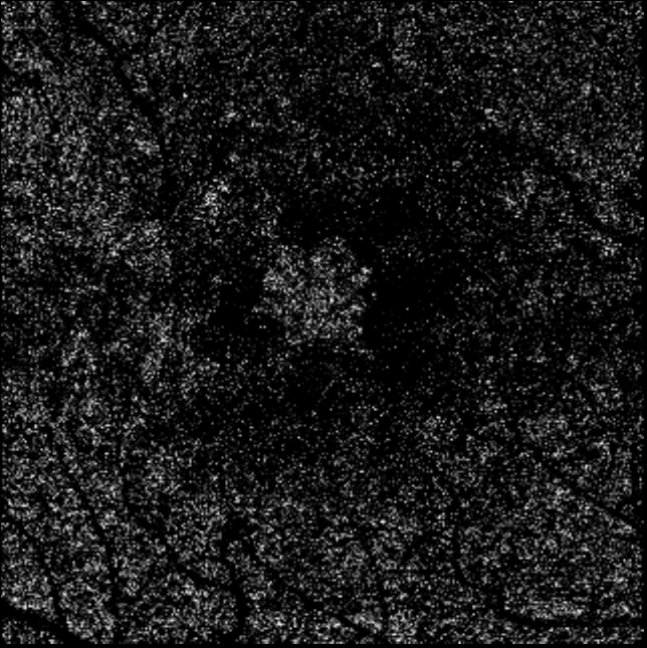

Supplement: S1 File — (ZIP) [file pone.0308292.s002.zip › Study eye/19.png]

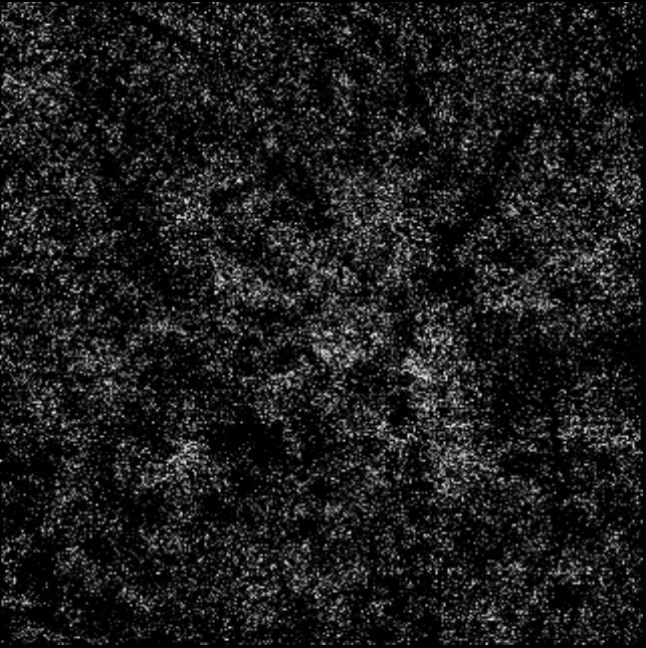

Supplement: S1 File — (ZIP) [file pone.0308292.s002.zip › Study eye/2.png]

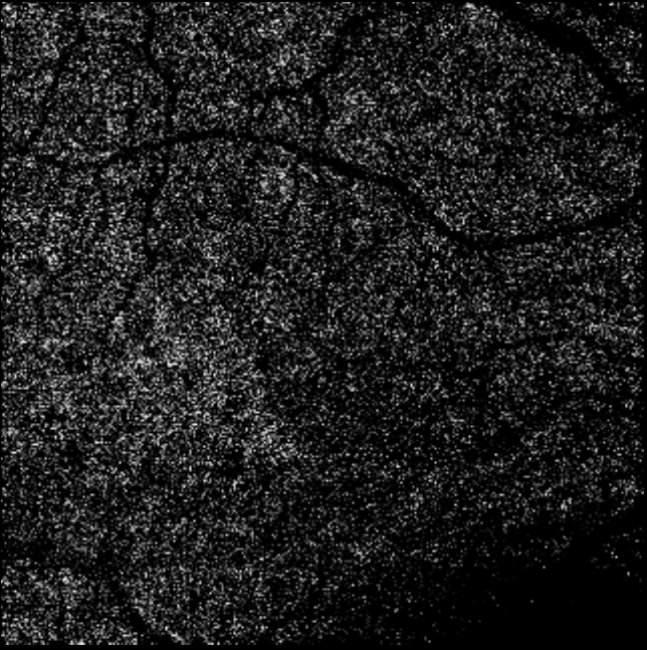

Supplement: S1 File — (ZIP) [file pone.0308292.s002.zip › Study eye/20.png]

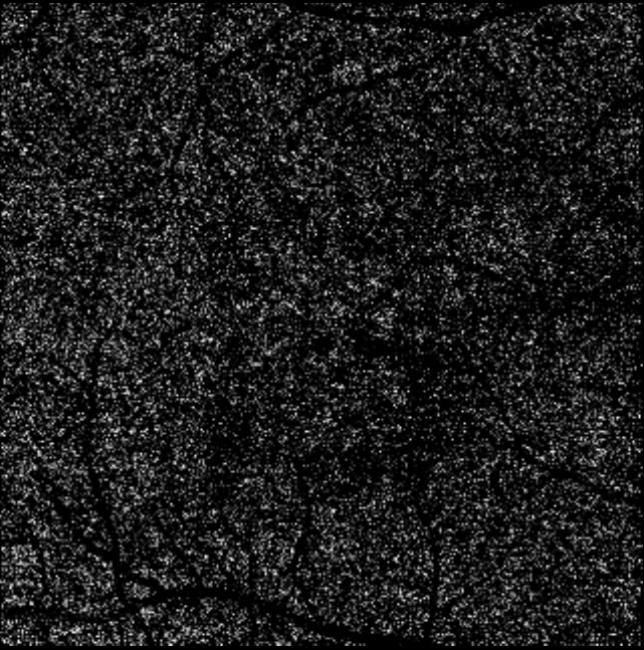

Supplement: S1 File — (ZIP) [file pone.0308292.s002.zip › Study eye/21.png]

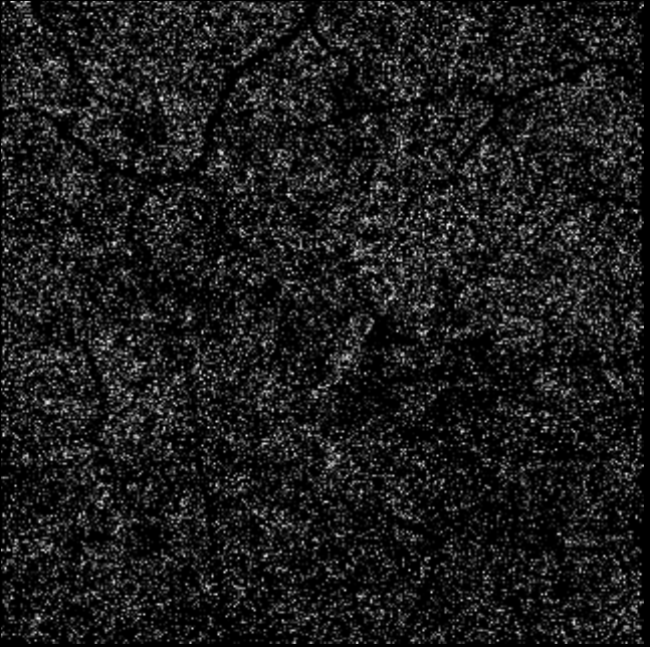

Supplement: S1 File — (ZIP) [file pone.0308292.s002.zip › Study eye/22.png]

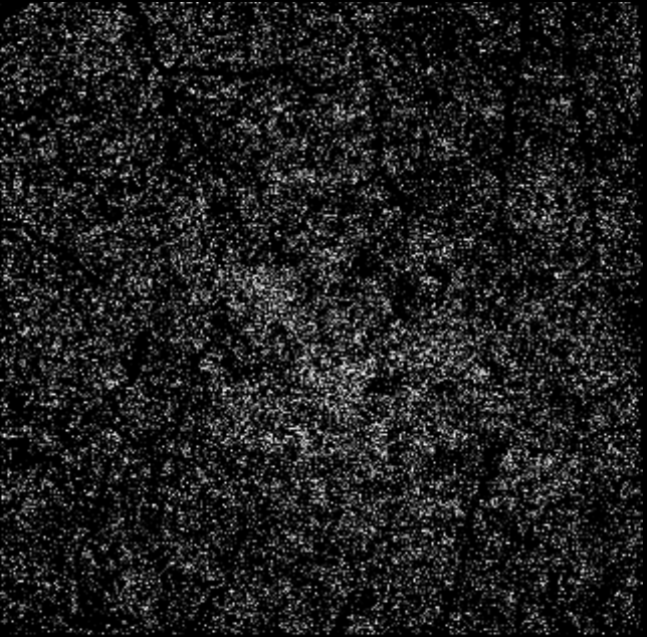

Supplement: S1 File — (ZIP) [file pone.0308292.s002.zip › Study eye/23.png]

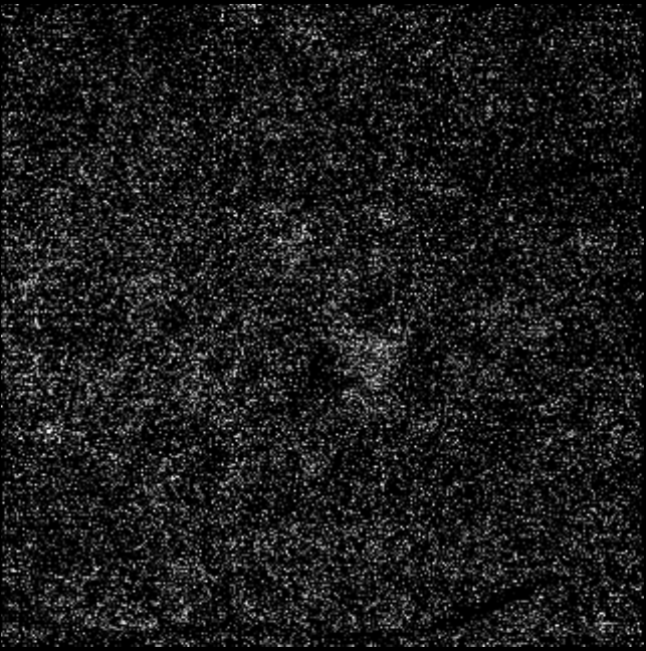

Supplement: S1 File — (ZIP) [file pone.0308292.s002.zip › Study eye/24.png]

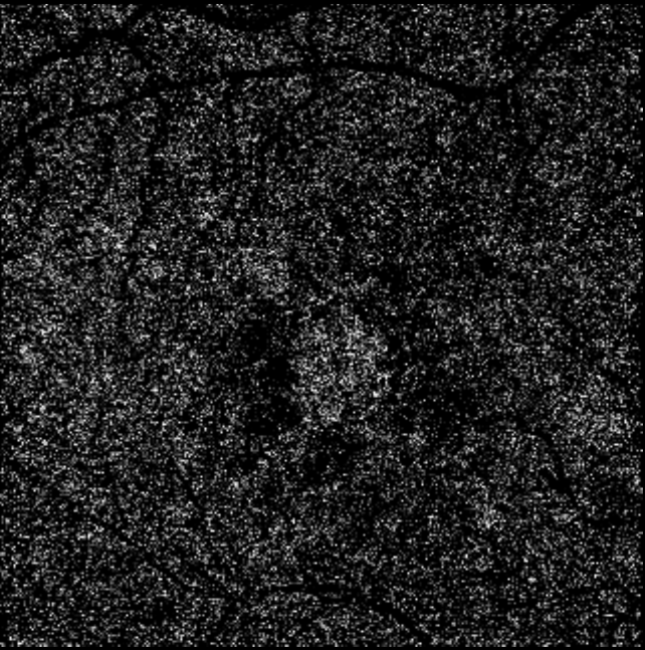

Supplement: S1 File — (ZIP) [file pone.0308292.s002.zip › Study eye/25.png]

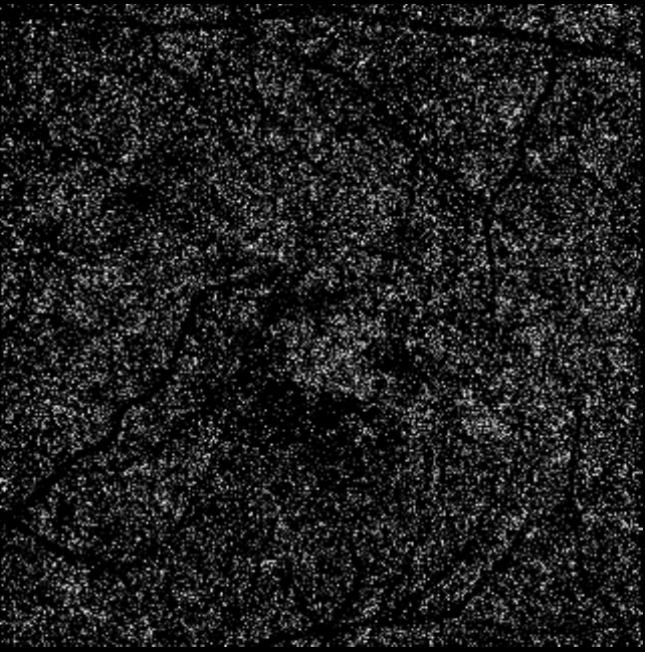

Supplement: S1 File — (ZIP) [file pone.0308292.s002.zip › Study eye/26.png]

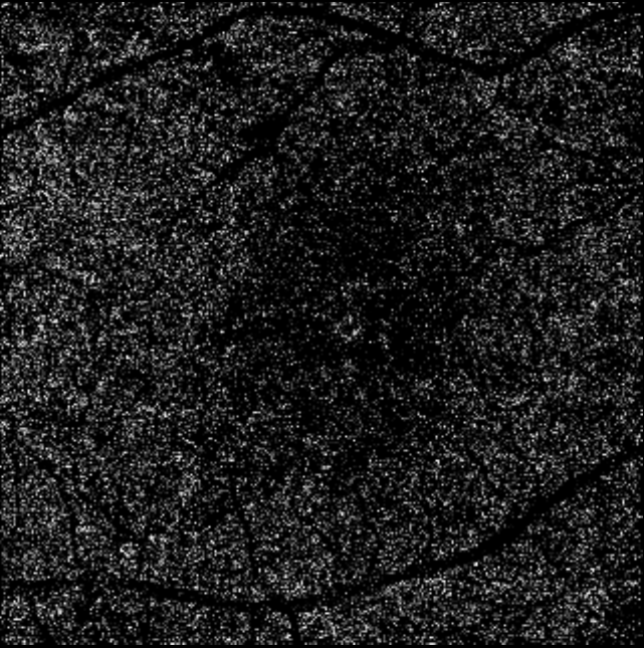

Supplement: S1 File — (ZIP) [file pone.0308292.s002.zip › Study eye/27.png]

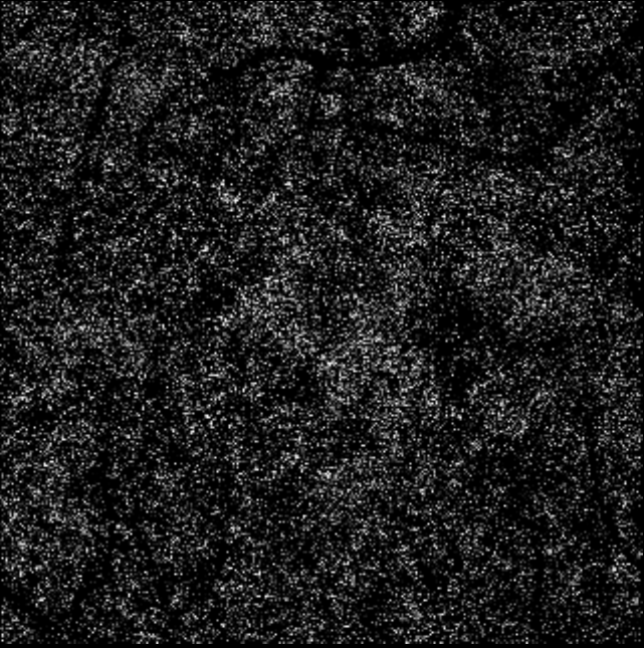

Supplement: S1 File — (ZIP) [file pone.0308292.s002.zip › Study eye/3.png]

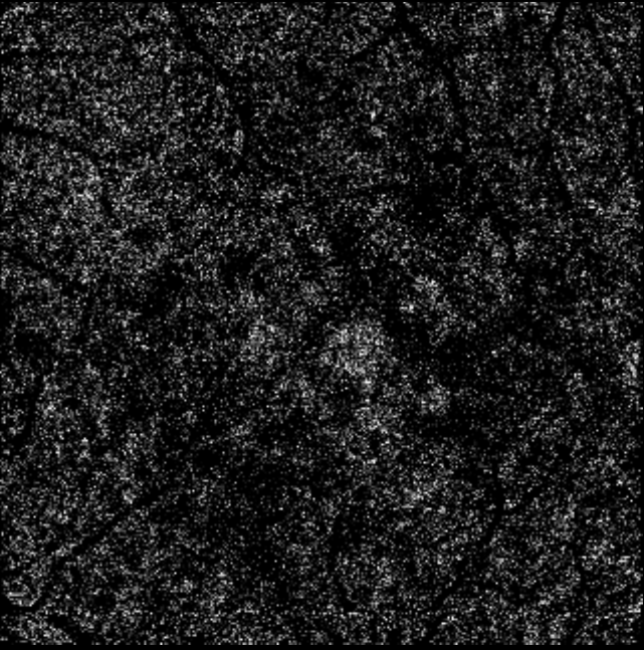

Supplement: S1 File — (ZIP) [file pone.0308292.s002.zip › Study eye/4.png]

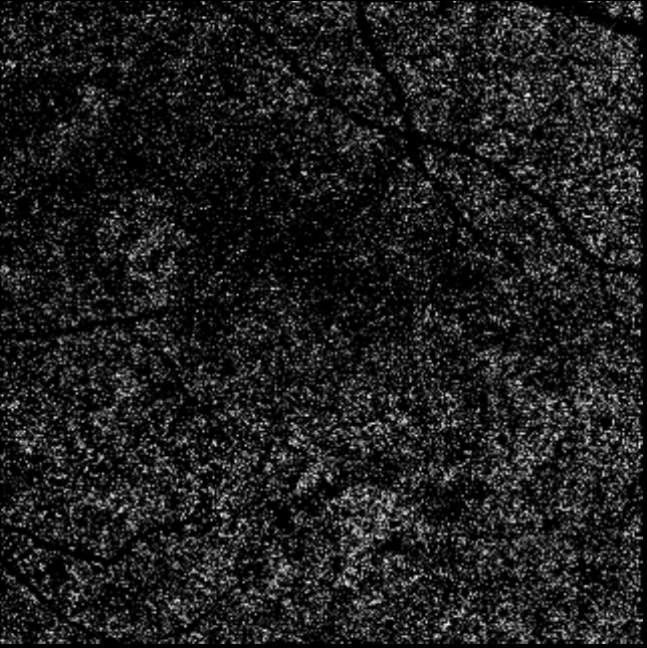

Supplement: S1 File — (ZIP) [file pone.0308292.s002.zip › Study eye/5.png]

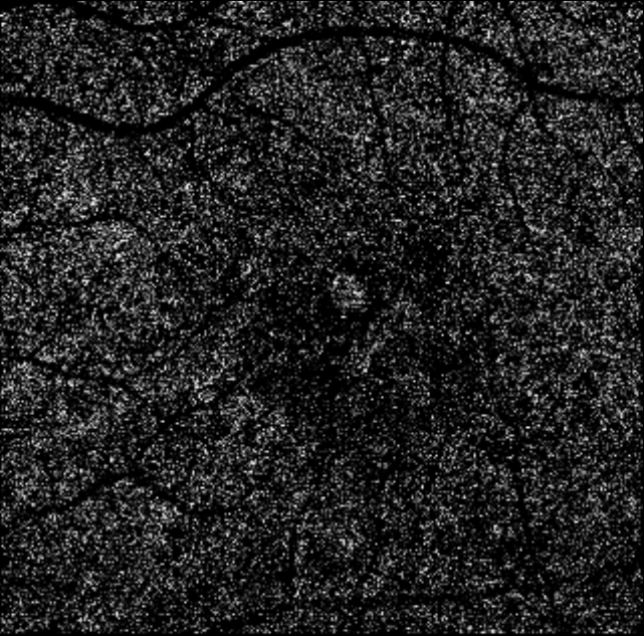

Supplement: S1 File — (ZIP) [file pone.0308292.s002.zip › Study eye/6.png]

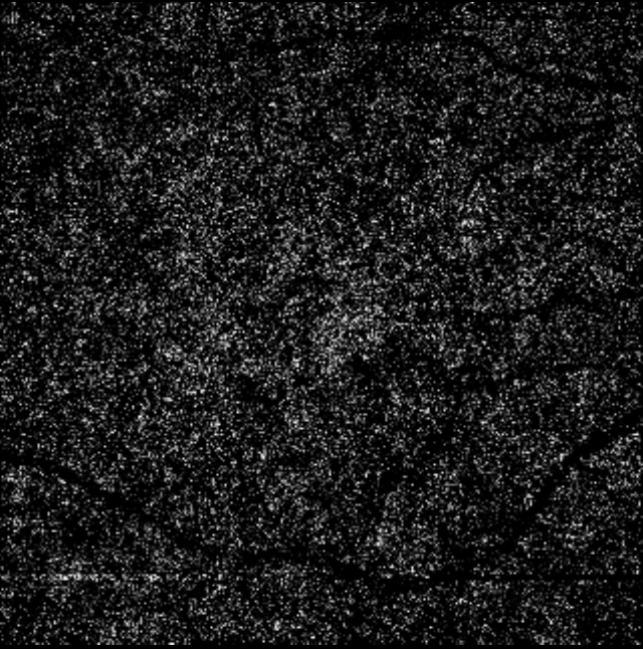

Supplement: S1 File — (ZIP) [file pone.0308292.s002.zip › Study eye/7.png]

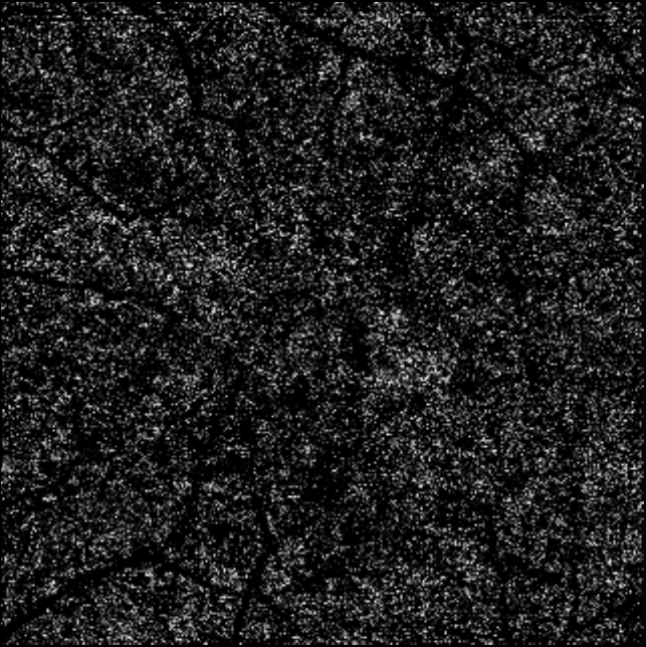

Supplement: S1 File — (ZIP) [file pone.0308292.s002.zip › Study eye/8.png]

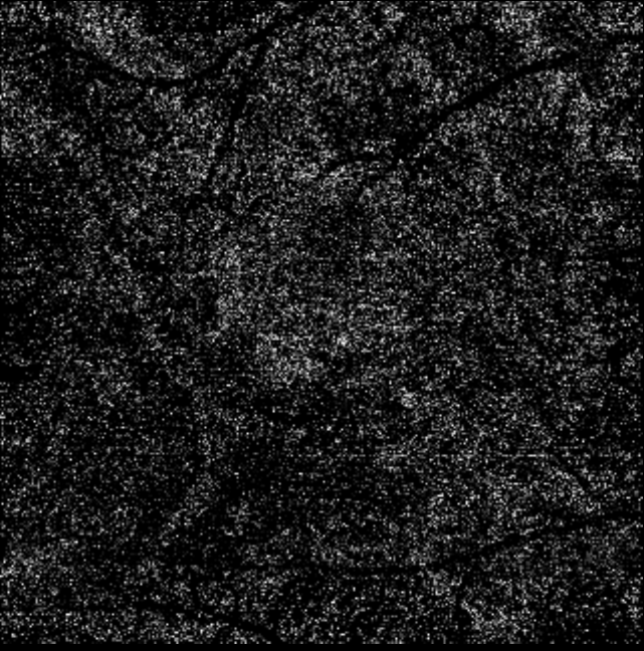

Supplement: S1 File — (ZIP) [file pone.0308292.s002.zip › Study eye/9.png]
